# Cross-species systems analysis distinguishes inflammatory remodeling from primary mucus secretory failure in inflammatory bowel disease

**DOI:** 10.3389/fimmu.2026.1813019

**Published:** 2026-06-09

**Authors:** Jessica Xhumari, Amanda Ojeda, Oluwamayowa S. Akinsuyi, Luiz F. W. Roesch

**Affiliations:** Department of Microbiology and Cell Science, Institute of Food and Agricultural Sciences, University of Florida, Gainesville, FL, United States

**Keywords:** inflammatory bowel disease (IBD), *MUC2*, mucin, mucosal inflammation, mucus barrier dysfunction

## Abstract

**Introduction:**

Intestinal mucus and epithelial barrier dysfunction are central features of inflammatory bowel disease (IBD), yet the regulatory mechanisms underlying mucus disruption remain inconsistent across individual studies. Anterior gradient 2 (*AGR2*) is an endoplasmic reticulum chaperone required for mucin folding, and its loss in mice causes primary mucus secretory failure and spontaneous colitis. Here, we integrated cross-cohort human transcriptomics with a genetic model of mucus secretory collapse to determine whether mucus dysfunction in IBD reflects intrinsic mucin-folding defects or secondary inflammatory remodeling.

**Methods:**

We performed a meta-analysis of 26 independent human colonic transcriptomic datasets comprising 1,302 active, untreated samples to derive a consensus mucus-barrier regulatory program in Crohn’s disease (CD) and ulcerative colitis (UC). Random-effects modeling identified differentially expressed genes, which were evaluated using gene set enrichment for biological processes relevant to mucus regulation. To provide a mechanistic benchmark for primary secretory failure, we generated an orthogonal colon proteome from *Agr2*^-/-^ mice.

**Results:**

Across human cohorts, 3,129 genes in CD and 3,729 in UC were differentially expressed, with substantial overlap between diseases. Both conditions showed coordinated upregulation of pathways linked to goblet-cell differentiation, mucin transcription, post-translational glycosylation, secretion, microbial sensing, and endoplasmic reticulum quality control. *MUC1*, *MUC4*, *MUC5AC*, and *MUC5B* were consistently upregulated in active IBD relative to healthy controls. *MUC2* was strongly elevated in CD. Glycosyltransferases, including *ST3GAL1* and *FUT8*, were upregulated, and secretion-associated immune regulators, such as *NOD2* and *CASP1*, increased, whereas the barrier lectin *ZG16* decreased. ER stress components *HSPA5*, *XBP1*, and *AGR2* were also upregulated compared with controls.

**Discussion:**

Cross-species comparison revealed convergence between IBD and *Agr2* deficiency in unfolded-protein response and microbial sensing pathways, including lipopolysaccharide and Toll-like receptor signaling. However, cytokine-driven regulatory programs prominent in IBD, including IL-6–STAT3 and IL-17 pathways, were not recapitulated in *Agr2*^-/-^ mice. These findings indicate that mucus barrier dysfunction in human IBD reflects inflammatory remodeling of epithelial programs rather than primary secretory collapse alone, while sharing conserved ER-stress and microbial response signatures. Integrating human and murine datasets identifies candidate pathways that may stabilize mucin folding, refine glycan composition, and preserve host-microbe spatial segregation in intestinal disease.

## Introduction

Inflammatory bowel disease (IBD) is a chronic inflammatory disorder of the gastrointestinal tract that manifests as Crohn’s disease (CD) or ulcerative colitis (UC). The pathology is characterized by the abnormal and prolonged activation of a T cell-mediated immune response aimed toward commensal microbes in the gut, compounded by genetic predisposition, environmental factors, and impaired epithelial barrier function. Current standard therapies target immune dysregulation through immunosuppressants or corticosteroids. However, the refractory rate for these treatments remains common (1), and surgical intervention is often still required. Although biomarkers have emerged from genetic, immunological, microbiome, and transcriptomic studies, most therapeutic strategies focus on downstream immune signaling rather than upstream epithelial processes that may initiate or sustain inflammation. Increasing attention has therefore shifted toward the gut barrier interface as a potential driver of disease pathogenesis.

The intestinal mucus layer represents the frontline barrier separating the microbiota from the epithelium and plays a central role in maintaining intestinal homeostasis (2). Mucin glycoproteins are secreted by goblet and epithelial cells and form a viscoelastic matrix that limits microbial and antigen interaction with the gut epithelial surface. Disruptions in goblet cell differentiation, mucin expression, or mucin glycosylation can compromise mucus structure and increase epithelial exposure to luminal contents (3). When the mucus barrier fails to maintain a safe distance between the microbiota and the epithelium, continuous exposure to antigens such as lipopolysaccharide (LPS) can drive chronic innate and adaptive immune activation (4). Consistent with this model, thinning of the mucus layer correlates with endoscopic disease severity in ulcerative colitis (5). Among the regulatory proteins governing mucus integrity, Anterior Gradient 2 (*Agr2*) has emerged as a pivotal endoplasmic reticulum (ER) chaperone required for the correct folding of the major secreted mucin MUC2. Loss of *Agr2* in mice results in defective mucus production and spontaneous colitis (6), and rare human mutations in *AGR2* can cause monogenic infantile IBD (7).

Various case-control studies have characterized the transcriptomic profiles of IBD patients, with multiple studies reporting alterations in mucin protein expression or related barrier genes (8, 9). However, considerable variability persists across findings, leaving the literature without a clear consensus on the mechanisms underlying mucus barrier disruption. Conflicting reports have been observed regarding the regulation of the major mucin gene *MUC2*, with some studies reporting decreased expression in IBD patients (10), increased expression (11), or no significant change (12). Inconsistencies may stem from differences in study design, patient demographics, geography, diet, lifestyle, and technical methods. Resolving these discrepancies is necessary not only to define robust molecular signatures but also to determine whether observed transcriptional changes reflect primary epithelial defects or secondary responses to inflammation. Meta-analysis of transcriptomic datasets provides a framework to identify conserved regulatory programs across heterogeneous cohorts by integrating independent studies and reducing study-specific noise. Nevertheless, transcriptomic measurements alone cannot distinguish whether altered gene expression represents causal epithelial dysfunction or adaptive remodeling, highlighting the need for orthogonal biological benchmarks at the protein and functional level.

Here, we tested the hypothesis that mucus barrier dysfunction observed in human IBD reflects either primary mucin secretory failure or inflammation-driven remodeling of epithelial programs. To address this question, we performed a cross-cohort meta-analysis of 26 independent colonic transcriptomic datasets to derive a consensus regulatory signature of mucus barrier disruption in CD and UC. We then compared these human transcriptional programs with an orthogonal colon proteome generated from *Agr2*^-/-^ mice, a model characterized by genetically defined collapse of mucin folding and secretion. By using *Agr2* deficiency as a mechanistic reference state for primary secretory failure, we sought to determine which regulatory pathways are shared with human disease and which are uniquely associated with inflammatory remodeling. This integrative approach moves beyond descriptive dataset consolidation to provide a systems-level framework for interpreting mucus barrier dysfunction in IBD and identifying pathways that support epithelial resilience and host-microbe spatial segregation.

## Results

### Cohort integration defines a consensus transcriptional program in active IBD

To determine whether mucus barrier dysfunction in inflammatory bowel disease reflects a conserved epithelial regulatory program, we first established a consensus transcriptional signature across independent human cohorts. A systematic search of the GEO database identified 571 potentially relevant datasets, which were screened using predefined inclusion criteria, including active disease, availability of healthy controls, and sufficient cohort size (≥ 4 samples per group). Studies composed exclusively of remission samples, drug-treated patients, or formalin-fixed paraffin-embedded tissue were excluded to minimize confounding effects related to therapy or RNA degradation. To avoid cohort redundancy, sample identifiers and metadata were cross-checked to ensure that each dataset represented a unique patient population.

**Figure 1:**
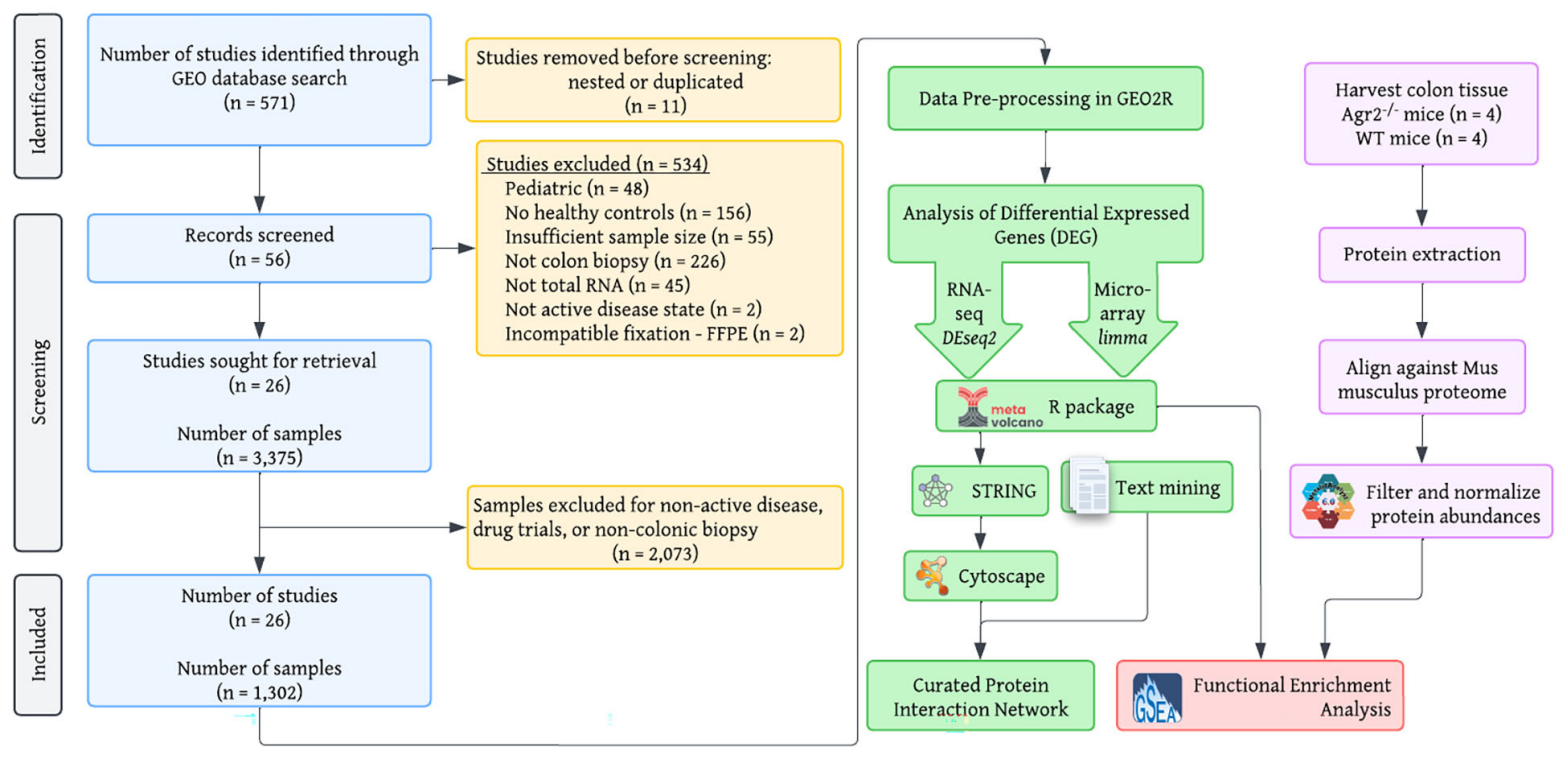
Cross-cohort integration defines a consensus transcriptional landscape of active inflammatory bowel disease. Selected transcriptomic datasets were pre-processed and analyzed for differentially expressed genes to detect enriched pathways relevant to mucus barrier function in Crohn’s disease (CD) and ulcerative colitis (UC) and construct a curated regulatory gene network with CD and UC transcriptomic expression profiles.

These filtering steps yielded 26 independent transcriptomic studies comprising 3,375 samples (**Table 1**). Restricting analyses to active, untreated disease resulted in a final dataset of 1,302 colonic biopsy samples used for comparative analyses of Crohn’s disease (CD) versus healthy controls and ulcerative colitis (UC) versus healthy controls (**Figure 1**). This design enabled evaluation of epithelial transcriptional programs specifically associated with active barrier dysfunction rather than treatment response or disease remission.

**Table 1:** Integrated human cohorts used to derive the consensus IBD mucus-barrier transcriptional signature.

#	Accession	Type	Platform	Country	HC	CD	UC
*1*	GSE10191	Microarray	Affymetrix GeneChip Human Genome U133 Plus 2.0 Array [CDF: Hs133P_Hs_REFSEQ_8]	USA	11	0	8
*2*	GSE10616	Microarray	Affymetrix GeneChip Human Genome U133 Plus 2.0 Array [CDF: Hs133P_Hs_REFSEQ_8]	USA	11	14	10
*3*	GSE13367	Microarray	[HG-U133_Plus_2] Affymetrix Human Genome U133 Plus 2.0 Array	Denmark	10	0	8
*4*	GSE165512	RNAseq	Illumina HiSeq 2500 (Homo sapiens)	Italy	31	37	40
*5*	GSE16879	Microarray	[HG-U133_Plus_2] Affymetrix Human Genome U133 Plus 2.0 Array	Belgium	6	19	24
*6*	GSE179285	Microarray	Agilent-014850 Whole Human Genome Microarray 4x44K G4112F (Probe Name version)	USA	23	14	23
*7*	GSE36807	Microarray	[HG-U133_Plus_2] Affymetrix Human Genome U133 Plus 2.0 Array	UK	7	13	15
*8*	GSE38713	Microarray	[HG-U133_Plus_2] Affymetrix Human Genome U133 Plus 2.0 Array	Spain	13	0	15
*9*	GSE48634	Microarray	Illumina HumanHT-12 V4.0 expression beadchip	UK	26	0	24
*10*	GSE52746	Microarray	[HG-U133_Plus_2] Affymetrix Human Genome U133 Plus 2.0 Array (custom CDF: HGU133Plus2_Hs_ENTREZG.cdf version 15.1.0)	Spain	17	10	0
*11*	GSE53306	Microarray	Illumina HumanHT-12 WG-DASL V4.0 R2 expression beadchip	USA	12	0	13
*12*	GSE65114	Microarray	[HuGene-2_0-st] Affymetrix Human Gene 2.0 ST Array [transcript (gene) version]	Ireland	12	0	16
*13*	GSE66207	RNAseq	Illumina HiSeq 2500 (Homo sapiens)	USA	13	20	0
*14*	GSE66407	Microarray	[HG-U219] Affymetrix Human Genome U219 Array (ENSG Brainarray CDF Version 18.0.0)	Denmark	80	35	61
*15*	GSE67106	Microarray	Gencode v15 lncrna.2 probe version1 Agilent Design ID (AMADID) 047718	Denmark	18	16	21
*16*	GSE6731	Microarray	[HG_U95Av2] Affymetrix Human Genome U95 Version 2 Array	USA	4	6	5
*17*	GSE73094	Microarray	Nanostring IBD Codeset	USA	26	83	0
*18*	GSE75214	Microarray	[HuGene-1_0-st] Affymetrix Human Gene 1.0 ST Array [transcript (gene) version]	Belgium	11	8	74
*19*	GSE83448	Microarray	CodeLink Human Whole Genome Array [DISCOVERY probe type]	Spain	14	19	0
*20*	GSE83687	RNA seq	Illumina HiSeq 2500 (Homo sapiens)	USA	49	12	30
*21*	GSE92415	Microarray	[HT_HG-U133_Plus_PM] Affymetrix HT HG-U133+ PM Array Plate	USA	21	0	53
*22*	GSE9452	Microarray	[HG-U133_Plus_2] Affymetrix Human Genome U133 Plus 2.0 Array	Denmark	5	0	8
*23*	GSE95095	Microarray	Illumina HumanHT-12 WG-DASL V4.0 R2 expression beadchip	China	12	24	0
*24*	GSE9686	Microarray	Affymetrix GeneChip Human Genome U133 Plus 2.0 Array [CDF: Hs133P_Hs_REFSEQ_8]	USA	8	11	5
*25*	GSE97012	Microarray	Agilent-028004 SurePrint G3 Human GE 8x60K Microarray (Feature Number version)	USA	27	4	14
*26*	GSE261086	RNA seq	Illumina HiSeq 2500 (Homo sapiens)	USA	8	15	0

Patient health status was categorized as Healthy Control (HC), Crohn’s disease (CD), or ulcerative colitis (UC) based on metadata classifications.

We next integrated differential expression results across cohorts using a random-effects meta-analytic framework to derive a consensus human IBD expression signature. This approach reduces study-specific variability arising from differences in population demographics, geographic origin, and sequencing platforms, thereby enabling the identification of regulatory programs consistently associated with disease. Establishing this unified transcriptional landscape provided the foundation for subsequent analyses aimed at distinguishing shared barrier-stress responses from mechanisms indicative of primary mucus secretory failure, which were evaluated through cross-species comparison with the *Agr2*^-/-^ mouse model.

### Human IBD exhibits coordinated transcriptional remodeling of mucus barrier pathways

To define how mucus barrier biology is altered in inflammatory bowel disease, we next examined transcriptional programs consistently dysregulated across integrated cohorts. Differential expression results from all datasets were combined using a random-effects meta-analytic framework implemented in MetaVolcano, enabling identification of genes reproducibly altered in Crohn’s disease (CD) and ulcerative colitis (UC) relative to healthy controls despite inter-study heterogeneity.

This analysis identified 3,129 differentially expressed genes (DEGs) in CD (2,686 upregulated and 443 downregulated) and 3,729 DEGs in UC (3,320 upregulated and 413 downregulated). A substantial proportion of these genes overlapped across diseases, with 1,835 shared DEGs representing 59% of CD-associated genes and 50% of UC-associated genes, indicating a conserved transcriptional response underlying IBD-associated barrier dysfunction. All tested genes are provided in **Supplementary Table 1** (CD) and **Supplementary Table 2** (UC).

Rather than isolated gene alterations, the shared transcriptional signature revealed coordinated regulation of biological processes central to mucus barrier maintenance. Genes encoding both gel-forming and membrane-bound mucins were consistently upregulated, including *MUC1*, *MUC4*, *MUC5AC*, and *MUC5B* in both diseases, while *MUC2* expression increased significantly in CD and trended toward upregulation in UC. These patterns suggest enhanced transcriptional activation of mucin biosynthesis programs during active disease.

In parallel, genes involved in microbial sensing and secretion-associated immune signaling were strongly upregulated. *CASP1*, a component of inflammasome-mediated epithelial defense, increased approximately threefold in CD and 2.5-fold in UC (p < 0.001), while *NOD2*, a bacterial pattern-recognition receptor linked to mucus secretion regulation, increased by 2.3-fold in both conditions (p < 0.001). Transcriptional regulators associated with epithelial adaptation were activated, including *HIF1A*, which promotes hypoxia-responsive mucin production, and *STAT1*, a mediator of interferon-driven epithelial remodeling.

Evidence of altered mucin processing was also observed through significant upregulation of glycosyltransferase genes. *ST3GAL1*, involved in terminal mucin sialylation, increased in CD (log_2_FC = 0.97, p < 0.001) and UC (log_2_FC = 1.29, p < 0.001), while *FUT8*, responsible for core fucosylation contributing to mucin stability, was elevated in UC (log_2_FC = 1.20, p < 0.001). Consistent with increased secretory demand, components of the endoplasmic reticulum stress response were elevated, including the unfolded protein response regulator *XBP1* and ER chaperone pathways linked to mucin folding.

Inflammatory cytokine programs known to influence epithelial barrier regulation were activated. *IL1B*, *IL6*, *IL17A*, and *IL33* were significantly upregulated in both CD and UC, whereas *IL10* enrichment was more prominent in UC. *IL1B* expression increased more than fourfold in both diseases, while *IL17A* exhibited strong induction in CD and UC, indicating activation of cytokine networks capable of driving epithelial remodeling and mucin transcription.

Together, these findings provide evidence that active IBD is characterized by coordinated transcriptional remodeling of mucus barrier biology involving simultaneous activation of mucin biosynthesis, post-translational modification, microbial sensing, inflammatory signaling, and endoplasmic reticulum stress pathways, rather than isolated dysregulation of individual genes.

### Regulatory network analysis reveals integrated epithelial and inflammatory control of mucus biology

**Figure 2:**
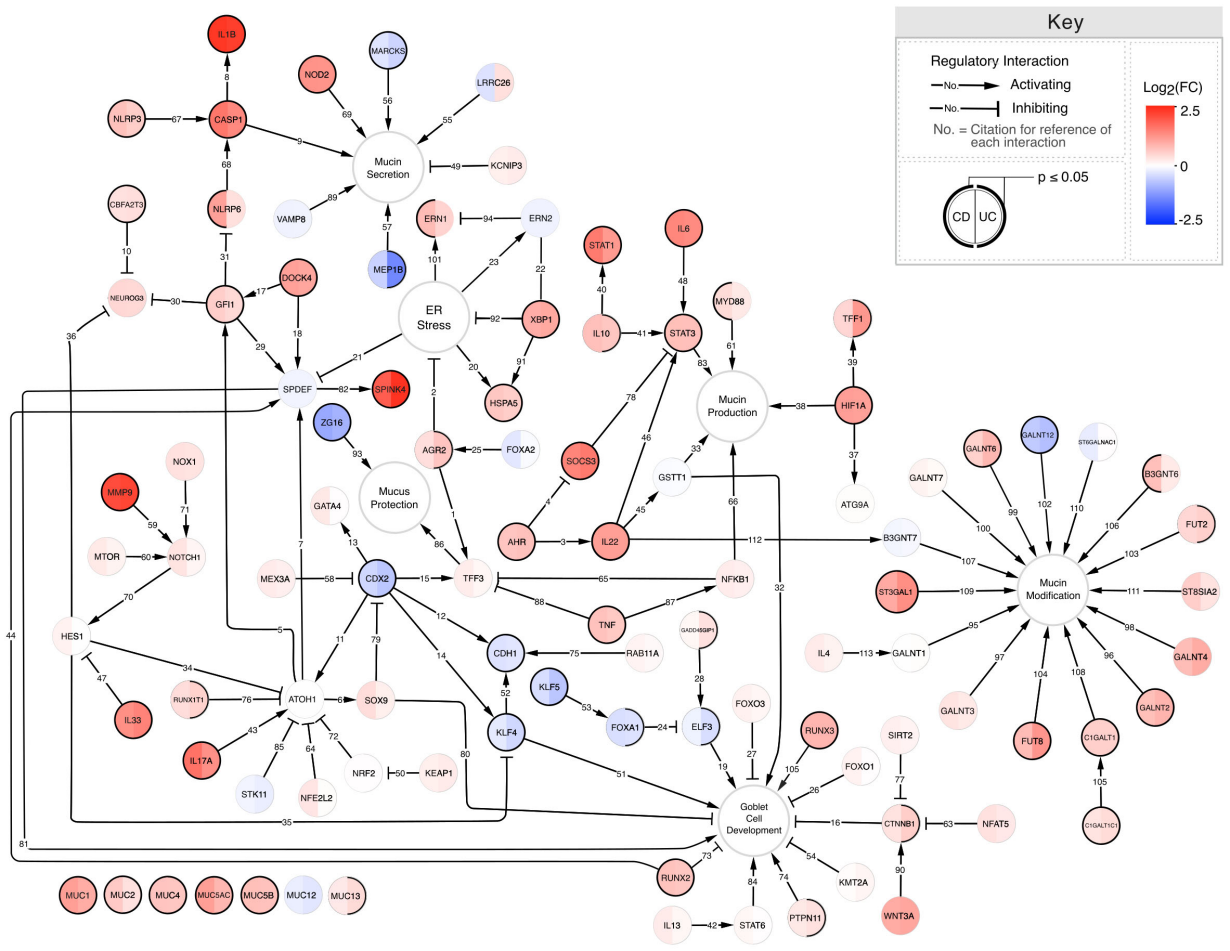
Regulatory network analysis reveals coordinated control of mucus barrier remodeling in inflammatory bowel disease. A comprehensive regulatory network was constructed to convey protein-protein interactions and mechanisms affecting mucin function and goblet cell development in active Crohn’s disease and ulcerative colitis. Relevant proteins and protein relationships in this network were first identified through literature findings. Activating relationships between proteins or between a protein and a biological function have an arrow (→) while inhibitory relationships have a blunt arrow (┴). Along each arrow is a citation number which corresponds to a study that has observed that activating or inhibiting relationship. This reference list can be found in **Supplementary References**. The log_2_ Fold Change of the differentially expressed genes obtained in our meta-analysis is represented in red (upregulated) or blue (downregulated). A black border around the node represents the statistical significance (p-value ≤ 0.05). The left side of each node conveys CD gene expression, and the right conveys UC.

Because differential expression alone does not reveal how mucus barrier pathways are coordinated during disease, we next asked whether the IBD transcriptional signature organizes into higher-order regulatory modules linking epithelial stress responses, immune signaling, and mucin biosynthesis. Regulatory network analysis revealed a modular organization of the mucus-barrier transcriptional program in both Crohn’s disease (CD) and ulcerative colitis (UC) (**Figure 2**). Citations corresponding to this figure can be found in the **Supplementary References**.

To examine these relationships, we constructed a curated interaction network linking differentially expressed genes through protein-protein and regulatory interactions previously supported by experimental studies and reported in the literature. Integration of transcriptomic expression data into this framework enabled visualization of regulatory connectivity underlying disease-associated transcriptional changes rather than isolated gene perturbations. Notably, these coordinated modules were not apparent from differential expression analysis alone but emerged only after incorporating regulatory relationships among genes.

Gene Set Enrichment Analysis of the curated mucus-barrier gene set confirmed coordinated regulation in both CD (NES = 1.45, p = 0.003) and UC (NES = 1.57, p < 0.001) (**Supplementary Figure 1**), indicating that mucus-associated genes shift collectively across independent cohorts.

Network organization identified several interconnected regulatory modules underlying mucus barrier remodeling. A mucin biosynthesis module linked upregulated gel-forming and membrane-bound mucins with transcriptional regulators of epithelial adaptation, including *HIF1A* and *STAT1*, suggesting integration of hypoxia-responsive and interferon-mediated signaling with mucus production programs. This organization indicates that increased mucin transcription occurs within a broader epithelial stress adaptation rather than as an isolated epithelial response.

A second module connected microbial sensing pathways with mucus secretion regulation. Upregulated nodes associated with *NOD2* and *CASP1* positioned bacterial recognition and inflammasome signaling within regulatory circuits controlling epithelial defense and mucus dynamics, supporting direct coordination between immune surveillance and barrier maintenance.

Post-translational modification pathways formed a third interconnected cluster in which glycosyltransferases, including *ST3GAL1* and *FUT8*, converged with unfolded protein response regulators such as *XBP1*. This arrangement suggests coupling between enhanced mucin biosynthesis and endoplasmic reticulum quality-control mechanisms required for proper protein folding and processing.

Inflammatory cytokine signaling served as a central organizing axis, bridging multiple modules. Cytokine-associated nodes linked immune activation with epithelial transcriptional regulation, connecting inflammatory signaling to mucin production, stress adaptation, and secretion pathways. Collectively, these relationships define an integrated regulatory architecture in which inflammatory signaling, epithelial stress responses, and mucin biosynthetic pathways converge to remodel mucus barrier biology in IBD.

### Cross-species comparison distinguishes shared barrier stress from primary secretory failure

To determine whether mucus barrier dysfunction in human IBD reflects primary epithelial secretory collapse or inflammation-driven remodeling, we compared the human transcriptional signature with a genetically defined model of mucin secretory failure. *Agr2*-knockout (*Agr2*^-/-^) mice exhibit defective MUC2 folding, severe endoplasmic reticulum (ER) stress, and collapse of the colonic mucus layer, thereby providing a mechanistic reference state for primary mucus secretory dysfunction. All *Agr2*^-/-^ mice also exhibited early signs of colitis with decreased weight compared to controls and symptoms of abnormal stool from soft to bloody diarrhea over a two week observation period. On the day of sample collection, female *Agr2*^-/-^ mice weighed 14.57 g on average compared with 20.2 g for female WT mice, whereas male *Agr2*^-/-^ mice weighed 11.7 g on average compared with 25.05 g for male WT mice.

**Figure 3:**
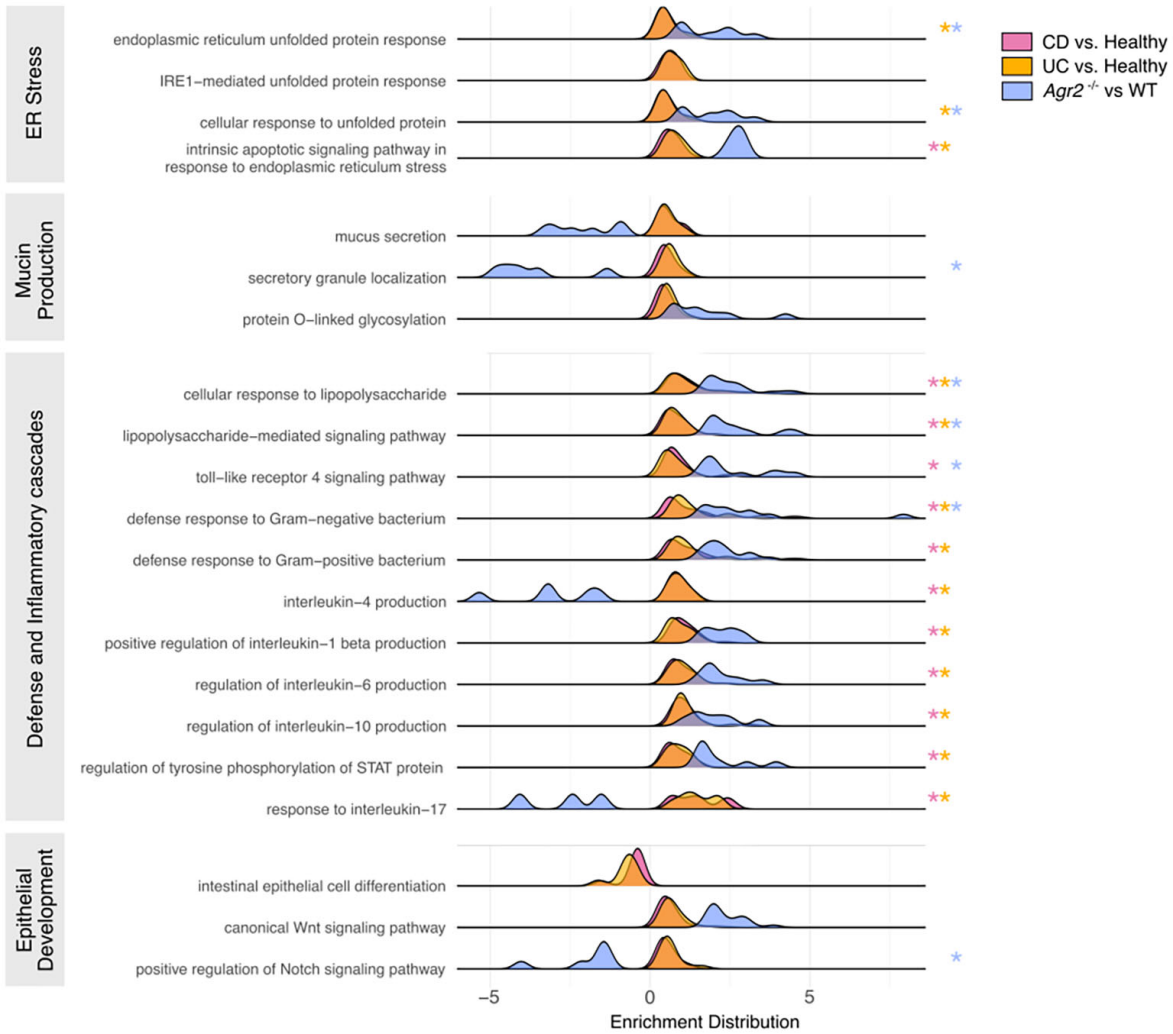
Cross-species comparison distinguishes shared barrier-stress responses from primary mucin secretory failure. In this plot, Gene Set Enrichment Analysis (GSEA) was performed on Reactome pathways relevant to mucus barrier function in Crohn’s disease (CD), ulcerative colitis (UC), and *Agr2* deficient mice. By ranking differentially expressed genes by log2(FC), GSEA identified if pathways were positively or negatively enriched. The ridges represent the distribution of genes within each pathway, with height indicating the number of genes sharing similar enrichment scores. Significantly positively or negatively enriched gene sets are presented with *p ≤ 0.05, highlighting key biological processes linked to mucus barrier regulation in CD, UC, and *Agr2*^-/-^.

We generated and analyzed the colon proteome of *Agr2*^-/-^ mice, identifying 2,281 proteins that were differentially abundant relative to wild-type controls. Total proteins tested can be found in **Supplementary Table 3**. Gene Set Enrichment Analysis (GSEA) was then used to compare pathway-level enrichment between human IBD datasets and the *Agr2*^-/-^ proteome, focusing on biological processes related to ER stress, microbial sensing, epithelial development, and mucin processing (**Figure 3**). Pathway-level comparison provides an appropriate framework for multi-omic analysis across species because biological processes are more conserved than individual feature changes.

Consistent with disruption of mucus barrier integrity in both conditions, ER stress and unfolded protein response pathways were enriched in ulcerative colitis and *Agr2*^-/-^ mice. Gene sets associated with the “endoplasmic reticulum unfolded protein response” were significantly enriched in UC (NES = 1.38, p = 0.024) and *Agr2*^-/-^ mice (NES = 1.49, p = 0.031), as were pathways related to the “cellular response to unfolded protein” (UC NES = 1.34, p = 0.035; *Agr2*^-/-^ NES = 1.48, p = 0.025). These shared signatures indicate convergence on ER stress as a common downstream consequence of mucus barrier disruption.

Microbial sensing pathways exhibited similar overlap. Gene sets associated with “cellular response to lipopolysaccharide” were strongly enriched in CD (NES = 1.98, p < 0.001), UC (NES = 2.06, p < 0.001), and *Agr2*^-/-^ mice (NES = 1.43, p = 0.022), alongside enrichment of “defense response to Gram-negative bacterium” and Toll-like receptor signaling pathways across all conditions. These findings suggest that microbial ligand recognition represents a shared response arising after barrier compromise, independent of the initiating mechanism.

Despite these similarities, key regulatory pathways differed markedly between human disease and *Agr2*-knockout. Cytokine-associated programs that were strongly enriched in IBD were largely absent in the *Agr2*^-/-^ model. Gene sets related to cytokine production and signaling, including IL-4, IL-1β, IL-6, IL-10, and IL-17 responses, were significantly enriched in CD and UC but not detected in *Agr2*^-/-^ mice. In particular, IL-6 and IL-17 signaling pathways showed strong enrichment in CD, indicating activation of inflammatory regulatory circuits not recapitulated by primary mucin-folding defects alone.

In contrast, pathways uniquely altered in *Agr2*^-/-^ reflected disruption of secretory maturation rather than inflammatory remodeling. Negative enrichment of “positive regulation of Notch signaling pathway” (NES = -1.57, p = 0.027) and altered “secretory granule localization” (NES = -1.69, p = 0.011) were observed only in *Agr2*^-/-^ mice, consistent with impaired goblet cell differentiation and vesicular trafficking associated with intrinsic secretory failure.

Together, these results indicate that human IBD and *Agr2*^-/-^ share molecular signatures associated with downstream barrier stress. In particular, ER stress activation and microbial sensing diverge in their upstream regulatory programs. The cytokine-driven transcriptional networks observed in IBD are not reproduced by primary mucin secretory collapse, indicating that mucus barrier dysfunction in human disease cannot be explained solely by intrinsic epithelial folding defects. Instead, the IBD transcriptional landscape reflects inflammatory remodeling of epithelial and mucus regulatory programs superimposed upon shared barrier-stress responses.

## Discussion

Despite evidence linking mucus barrier dysfunction to IBD progression (13, 14), conflicting observations regarding mucus-associated gene regulation have made it difficult to define a unified disease mechanism. In this study, we addressed this uncertainty by testing whether mucus barrier disruption in human IBD reflects primary epithelial secretory failure or inflammation-driven remodeling of epithelial programs. By integrating cross-cohort human transcriptomics with a mechanistic reference model of mucin-folding deficiency, we provided evidence that, while IBD shares downstream barrier-stress responses with *Agr2*^-/-^, including endoplasmic reticulum (ER) stress and microbial sensing, the transcriptional landscape of human disease is distinguished by prominent cytokine-driven regulatory programs that are not reproduced by primary mucus secretory collapse. These findings indicate that mucus dysfunction in IBD reflects inflammatory remodeling superimposed upon conserved barrier-stress responses rather than intrinsic failure of mucin secretion alone.

Our transcriptomic meta-analysis of IBD colon samples revealed changes in gene expression associated with epithelial cell fate of mucus-secreting goblet cell in the colon epithelium. The transcription factor *RUNX2* gene expression was significantly increased, but the expression of its target, *SPDEF* (15), remained unperturbed, possibly indicating SPDEF-independent regulation of differentiation. Furthermore, *HIF1A*, a key regulator of epithelial adaptation to hypoxic conditions and mucin expression (16, 17), was elevated in both diseases, suggesting a shift toward stress-induced goblet cell differentiation. Moreover, *KLF5* is involved in goblet cell proliferation (18) and was decreased in CD and UC. It is also important to note that master regulation of goblet cell development via NOTCH signaling was not significantly altered in our compiled analysis despite an activator, *MMP9* (19), exhibiting upregulation. The simultaneous overactivation of goblet cell proliferation and differentiation genes with the depletion of goblet cells in UC (13) may represent a compensatory response to ongoing cellular loss. This may result in immature or dysfunctional goblet cells and ultimately compromise the mucus barrier.

At the transcriptional level, mucin biosynthesis programs were broadly activated. Upregulation of *MUC1*, *MUC4*, *MUC5AC*, and *MUC5B*, with disease-dependent increases in *MUC2*, helps reconcile longstanding discrepancies regarding mucin expression in IBD (20). Increased mucin transcription does not necessarily imply improved barrier function; excessive mucin synthesis can overwhelm ER folding capacity, leading to the accumulation of aberrant mucins and secretory stress (21). Our findings therefore support a model in which enhanced mucin transcription represents a compensatory response to barrier injury rather than restoration of normal mucus function.

Inflammatory cascades influencing *MUC* transcription were also observed. *STAT3* has been characterized as a transcriptional activator of *MUC1* expression (22), and its gene expression was heightened in CD and UC. The activation of *STAT3* is driven by Interleukin-6 and Interleukin-10, which also presented upregulated gene expression and have significantly enriched signaling pathways in both conditions (23, 24). Although *SOCS3*, a negative regulator of *STAT3* that inhibits IL-6 signaling, is also upregulated, IL-10 supports *STAT3* activation (25, 26). In parallel, IL-4 gene set pathways were positively enriched and are known to induce goblet cell hyperplasia and enhance mucin gene expression through *STAT6*, supporting secretory function in inflamed mucosal tissues (27). IL-1β programs were also enriched and can augment *MUC2* transcription and stimulate rapid mucus release through NF-κB and inflammasome pathways in epithelial cells (28). IL-17 pathways were likewise enriched and can increase mucin transcription and goblet differentiation via NF-κB/MAPK-dependent signaling, with synergistic effects alongside IL-1β during barrier stress (29). Another potential activator of *MUC5AC* transcription may be *HIF-1α*, Hypoxia-Inducible Factor 1-Alpha. Hypoxia within the epithelium may result from insufficient transepithelial oxygenation or vascularization that may arise from hyperplasia exceeding neovascularization (30). These pathways suggest that mucin overexpression in IBD arises from inflammatory and metabolic signaling rather than intrinsic epithelial secretory defects.

After translation, mucins undergo extensive post-translational modifications, such as glycosylation, which define their behavioral properties and their interactions with the gut environment. O-linked glycosylation of mucins is a complex process involving the attachment of various sugar moieties to serine or threonine residues of mucins, a process that influences mucus permeability, viscoelasticity, hydration, and resistance to proteolytic degradation (31, 32). Several key mucin glycosyltransferases were differentially expressed, such as *GALNT2* and *GALNT6*, which were overexpressed, while *GALNT12* was decreased in both diseases. Similarly, *FUT8*, another glycosyltransferase gene responsible for core fucosylation, was significantly elevated in UC. The altered regulation of these glycotransferase genes could contribute to the aberrant glycan profile previously observed in ulcerative colitis (14), which can compromise mucin barrier function. Additionally, the sialylation of mucins is critical for resisting bacterial degradation (33); however, in ulcerative colitis, higher levels of sialic acid have been observed in rectal mucin (34). A mucin-relevant sialyltransferase, *ST6GALNAC1*, was not perturbed in CD or UC. However, *ST3GAL1* known to mediate mucin sialylation in the lungs, was overexpressed in both CD and UC (35). This may reflect an attempt at counterbalance due to increased sialidase activity or mucin turnover, highlighting dynamic regulation of glycan composition during disease.

Furthermore, mucus secretion is a highly regulated process that supports continuous replenishment of the intestinal barrier while responding to microbial and inflammatory cues. Several key genes involved in mucus secretion were significantly altered in IBD, which could lead to disruptions in goblet cell exocytosis and mucosal defense. *NOD2* expression was increased in Crohn’s disease and it encodes a sensor for bacterial peptidoglycan, which is already well recognized for its immunological role in CD; however, more recently, it has also been found to regulate mucus secretion (36). Similarly, *CASP1*, a central component of the inflammasome that is implicated in mucin secretion (37) was strongly overexpressed in CD and UC. In contrast, *MEP1B* was downregulated in UC, which could influence mucin processing or detachment. This metalloendopeptidase is sensitive to microbial signaling as that is when it is shed into its soluble form, allowing it to cleave MUC2 proteins (38, 39). *NOD2*, *CASP1*, and *MEP1B* regulation in IBD reveals the intimate connection between active immune surveillance and mucus secretion, which can determine the function of the barrier irrespective of mucin assembly. Beyond secretion, the mucus layer also serves as a protective barrier by trapping and neutralizing microbes. *ZG16*, a lectin that plays a key role in aggregating bacteria and preventing their penetration into the mucus layer (40), was significantly underexpressed, potentially compromising the ability of mucus to trap and neutralize pathogens. These results support previous findings that colitis exhibits both excessive mucus secretion responses to microbial stimulation and a breakdown of protective mechanisms that maintain mucus integrity (14, 41).

We further characterized the endoplasmic reticulum (ER) response in IBD since it has been linked to altered secretion, mucin folding, and glycan processing in mouse studies (42). Mucin proteins are translated in the rough ER and then in the lumen, they are folded, polymerized by disulfide bonds, and prepared for glycosylation in the Golgi apparatus. Misfolded mucins can aggregate intracellularly, leading to ER stress and triggering apoptosis in severe cases (43, 44). This can elicit the unfolded protein response (UPR) to restore proteostasis and maintain secretory function. Consistent with this, both CD and UC showed increased expression of canonical UPR components such as the chaperone *HSPA5* (45), transcription factor *XBP1*, and mucin-folding *AGR2*. *AGR2* expression is induced by ER stress and supports secretory homeostasis in goblet and Paneth cells by catalyzing essential disulfide bonds on mucins for secretion (46). Because *AGR2* promotes proper mucin folding and secretion (6), its upregulation likely reflects compensatory attempts to maintain proteostasis rather than evidence of primary *Agr2*^-/-^ in most IBD cases.

Cross-species comparison with *Agr2*^-/-^ mice provided mechanistic insight into these observations. As expected, the *Agr2*^-/-^ model showed strong ER stress and unfolded protein response activation, together with enrichment of microbial sensing pathways, consistent with barrier collapse. Similar overlap in LPS response and Toll-like receptor signaling between *Agr2*^-/-^ mice and IBD indicates that microbial recognition pathways represent shared downstream consequences of mucus disruption. However, cytokine-driven regulatory programs prominent in human IBD, including IL-6/STAT3 and IL-17 signaling, were not recapitulated in *Agr2*^-/-^. Instead, the mice displayed alterations related to secretory maturation and granule localization, reflecting intrinsic defects in mucin processing. These differences demonstrate that inflammatory signaling networks observed in IBD arise independently of primary mucin-folding failure. Notably, *Agr2*^-/-^ is also associated with the expansion of adherent-invasive *E. coli*, a pathobiont linked to Crohn’s disease (47), supporting the hypothesis that microbial dysbiosis and ER stress may reinforce each other following barrier disruption.

While our findings provide new insights into mucus barrier dysregulation in IBD, several limitations should be acknowledged. First, inter-study heterogeneity is an inherent challenge in meta-analyses of public datasets, including differences in cohort demographics, disease severity, biopsy handling, and sequencing platforms. Although we applied a random-effects model through MetaVolcano to mitigate between-study differences, residual variability likely persists and is exacerbated by missing metadata on biopsy sublocation and cell-type composition in bulk tissue. Additionally, the *Agr2*^-/-^ represents a single loss-of-function model in a controlled environment which captures aspects of IBD biology but does not fully recapitulate the inflammatory complexity of human IBD. Furthermore, our approach relied on cross-species comparisons of transcriptomic and proteomic datasets which enables pathway-level integration but direct features of genes and proteins are not directly equivalent as they may be influenced by post-transcriptional regulation and protein turnover. Species-specific differences in intestinal physiology, microbiota, and immune response may limit direct translatability to human IBD, and the global knockout limits disentangling other effects from systemic immune or microbial influences. Future studies combining inhibition of IL-6/STAT3 or IL-17 signaling and patient-derived organoids with *AGR2* perturbation will help validate whether *AGR2*-associated ER stress represents an intrinsic secretory defect or downstream consequence of inflammatory remodeling in IBD. From a translational perspective, these findings suggest that pathways regulating ER stress, glycosylation, and cytokine signaling may be therapeutic targets for restoring mucus barrier integrity. Modulation of unfolded protein response pathways and interventions targeting aberrant glycosylation patterns may help restore epithelial secretory function and barrier homeostasis.

In summary, our integrative analysis defines a consensus transcriptional framework linking mucin biosynthesis, glycosylation, ER stress, microbial sensing, and inflammatory signaling in IBD. While *Agr2*^-/-^ recapitulates shared barrier-stress responses, the absence of cytokine-driven regulatory remodeling in this model indicates that mucus dysfunction in human IBD is not explained solely by primary secretory failure. Instead, inflammatory signaling reshapes epithelial and mucus regulatory programs, producing a remodeled barrier state. These findings refine the conceptual framework of mucus-barrier dysfunction in IBD and identify pathways that may support mucin folding, trafficking, glycan composition, and epithelial proteostasis as potential targets for restoring mucosal barrier integrity.

## Materials and methods

### Study selection and data acquisition

We conducted a comprehensive search of transcriptomic datasets related to inflammatory bowel disease using the Gene Expression Omnibus (GEO) database. The study design and selection process are illustrated in **Figure 1**. We queried GEO using the terms [(“Inflammatory Bowel Disease”) OR (“Crohn’s disease”) OR (“ulcerative colitis”)] AND [“Homo sapiens” (Organism)] AND [gse (Filter)]. We established specific inclusion criteria to ensure a focused and clinically relevant dataset. Studies were included if they (i) employed microarray or RNA-seq platforms; (ii) examined colon biopsies from non-pediatric patients with active IBD (Crohn’s disease [CD] or Ulcerative Colitis [UC]); (iii) included healthy control samples; (iv) had at least 4 samples per group; and (v) utilized total RNA from fresh, non-formalin-fixed, paraffin-embedded tissue. We excluded duplicates, subsets of larger series, and studies involving patient samples undergoing experimental therapeutics. After applying these criteria and filtering for independent datasets, our final set of studies focused on active disease without confounding interventions.

All datasets used in this analysis are available in the Gene Expression Omnibus repository, https://www.ncbi.nlm.nih.gov/gds/, under the following accession numbers: GSE10191, GSE10616, GSE13367, GSE165512, GSE16879, GSE179285, GSE36807, GSE38713, GSE48634, GSE52746, GSE53306, GSE65114, GSE66207, GSE66407, GSE67106, GSE6731, GSE73094, GSE75214, GSE83448, GSE83687, GSE92415, GSE9452, GSE95095, GSE9686, GSE97012, GSE261086.

### Data processing and differential expression analysis

Each dataset was pre-processed to ensure consistency and comparability. Microarray data were normalized using quantile normalization and log transformation as implemented in GEO2R (https://www.ncbi.nlm.nih.gov/geo/geo2r/), and raw data were imported into R (version 4.4.3) using the GEOquery package (version 2.66.0). Differential expression for microarray datasets was assessed using the *limma* package (version 3.54.0) which is well-suited for continuous intensity data after normalization. In contrast, since RNA-seq datasets produce discrete count data, they were normalized and analyzed using the *DESeq2* package (version 1.44.0). In both cases, multiple-testing correction (Benjamini-Hochberg) was applied to control the false discovery rate, and consistent thresholds for statistical significance were used across platforms to ensure comparability.

To facilitate integration across platforms, we mapped all probes or genes to a common set of Entrez Gene identifiers using the provided annotation files. Unmapped or non-annotated features were excluded. For genes represented by multiple probes, we averaged their expression values to obtain a single representative measurement. We applied a significance threshold of p < 0.05, adjusted for multiple testing as appropriate.

### Meta-analysis

To integrate differential expression results across multiple studies, we employed the MetaVolcanoR package (version 1.0.1) in R which enables meta-analysis of differentially expressed genes (DEGs) while accounting for inter-study heterogeneity. For the cross-species comparison, this study used a pathway-level strategy conceptually consistent with multi-omics enrichment frameworks where transcriptomic and proteomic datasets are compared through shared pathway annotations rather than individual features (48). However, because the present analysis compared human disease transcriptomes with a murine proteomic model of primary mucus secretory failure, we did not statistically merge transcriptomic and proteomic p-values or effect sizes into a single integrated metric. Shared enrichment was therefore interpreted conservatively as convergence at the biological-process level rather than direct correspondence between individual transcripts and proteins. As input, we used differential expression results from multiple independent studies, each containing gene symbols, log_2_ fold changes (log_2_ FC), p-values, and confidence intervals. These datasets were first harmonized by extracting common gene symbols across studies and filtering for a predefined list of target genes related to mucosal biology and inflammation. We utilized the random effects modeling (REM) strategy because it accounts for both within-study and between-study variance, making it well-suited for datasets derived from heterogeneous experimental conditions, such as differences in sample sizes, platforms (RNA-seq vs. microarray), or population cohorts. The rem_mv() function was used to implement REM, which summarizes gene fold changes while incorporating confidence intervals to account for study variability. The rem_mv() function was run using package default parameters. To identify consistently dysregulated genes, we applied a threshold of p-value < 0.05 and absolute meta-log_2_ fold change (effect size) > 0.58, corresponding to a 1.5-fold change. This threshold balances biological relevance and statistical robustness and is widely used in transcriptomic meta-analyses to capture moderate and functionally meaningful expression changes across heterogeneous human datasets. This approach provides a systematic and statistically rigorous method for identifying consistently perturbed genes across multiple transcriptomic datasets in inflammatory bowel disease.

### Gene set enrichment analysis

To gain insight into how gene expression profiles impact mucus barrier function in CD and UC, we performed Gene Set Enrichment Analysis (GSEA) using the clusterProfiler (version 4.12.6) and fgsea R packages (version 1.30.0). GSEA assesses whether predefined gene sets exhibit significant enrichment at either the top (upregulated) or bottom (downregulated) of a ranked list of DEGs, allowing for identifying biological processes associated with mucus barrier regulation. Differential expression results, including gene symbols, log_2_ fold changes (log_2_ FC), and p-values, were obtained from MetaVolcano meta-analysis results. To ensure compatibility with functional annotation databases, gene symbols were converted to Entrez IDs using the bitr() function from org.Hs.eg.db (version 3.19.1), removing unmapped and duplicate entries. Genes were then ranked by log_2_ fold change, with the highest positive values placed at the top of the list, facilitating enrichment detection of both upregulated and downregulated pathways. We applied GSEA using gseGO(), specifying biological process (BP) Gene Ontology (GO) terms, with parameters set to minGSSize = 15, maxGSSize = 500, and pvalueCutoff = 0.05. Enrichment significance was determined using normalized enrichment scores (NES), which account for gene set size, and adjusted p-values (q-values), computed using Benjamini-Hochberg multiple testing correction.

### Curated regulatory network

A regulatory network was constructed to elucidate protein-protein interactions and regulatory mechanisms underlying mucus barrier dysfunction. Candidate nodes were curated from published literature based on predefined inclusion criteria including documented involvement in mucus barrier maintenance, goblet cell biology, mucin synthesis/secretion, epithelial integrity, ER stress, or inflammatory regulation of the intestinal epithelium as well as our prior work (49). Candidate interactions were then evaluated using a tiered evidence framework. Experimentally supported interactions in STRING were used as an initial reference point for protein-protein connectivity, but interaction directionality was assigned only when supported by primary literature describing activating, inhibitory, or regulatory effects in intestinal epithelial, goblet cell, mucin-related, or closely related inflammatory contexts. When multiple studies reported the same interaction, priority was given to primary experimental studies, disease or tissue relevant models, and more recent publications. Interactions with contradictory directionality were included only when the balance of evidence supported a consistent regulatory relationship across multiple studies, otherwise, the edge was excluded from the final network. Based on the referenced studies, each interaction was categorized as activating or inhibitory, with citation numbers along the edges. To incorporate transcriptomic expression data, the log_2_ FC and p-values obtained from our MetaVolcano REM meta-analysis were overlaid onto the network.

Visualization and comparative expression mapping between CD vs. control and UC vs. control were performed using Cytoscape, allowing for the side-by-side representation of disease-specific regulatory dynamics. The resulting network visualizes regulatory links between genes and their encoded proteins, aligning inflammation-associated gene expression with mucus barrier processes in active CD and UC.

### Animal care

All animal experiments were approved by and conducted in accordance with the Institutional Animal Care and Use Committee at the University of Florida (IACUC202300000249). B6.129S4(FVB)-*Agr2*<tm1.2Erle>/J mice were purchased from Jackson Laboratory, and WT and *Agr2*-/- mice were bred by the UF Rodent Models Breeding Core. Mice were housed in a 12-hour light-dark cycle at a controlled temperature (20-25 °C) and humidity (31-67%), with free access to a standard diet. Autoclaved water or SLIP-treated water was provided ad libitum. Health and well-being were monitored daily including weight check and stool consistency observations two weeks prior to sample collection, and mice were euthanized according to animal care guidelines.

### *Agr2*^-/-^ breeding and genotyping

The generation of *Agr2* knockout mice has been described previously (6). Briefly, the heterozygous knockouts purchased from Jackson Laboratory (B6.129S4(FVB)-*Agr2*<tm1.2Erle>/J, strain: Strain #:025630) were interbred to produce experimental homozygous KO and wild type littermate control mice. Genotyping for *Agr2*^-/-^ mice was performed by PCR using tail DNA and a set of 20629 (5′- GGT TTG GGC CTG AAA CTC TG -3′), 20630 (5′- ACC ATC AAG GGT CTG TTG CT -3′), and 20631 (5′- GGC CAT GGG TAC CTT TAG TG -3′) primers. The expected PCR product for the mutant is a single band of 400 bp. For heterozygous, it is two bands, one of 252 bp and another of 400 bp. Wild-type mice produce a single band of 252 bp.

### Proteomic analysis of *Agr2*^-/-^ vs WT

A total of 8 mice were used in this study, including wildtype (7 weeks) and *Agr2*^-/-^ (9 ± 2 weeks) (n=4 per group). In all mice subjects, a 1 cm segment was cut from the distal colon, which is often the most affected region in established murine colitis models (50, 51). Samples were flash frozen in liquid nitrogen and stored at -80°C. Tissue samples were processed by the University of Florida Mass Spectrometry Research and Education Center using tissue digestion and mass spectrometry. Proteins were extracted from colon and spleen tissues using the EasyPep™ MS Sample Prep Kit (Thermo Fisher Scientific) and quantified with the Qubit system. Each sample was prepared with an equimolar dilution of 100 μg total protein for digestion using the Rapid-Digestion Trypsin/Lys-C Kit (Promega) according to the manufacturer’s instructions. Samples were incubated with 0.1 M dithiothreitol in 100 mM ammonium bicarbonate at 56°C for 30 minutes, followed by 0.54 μL of 55 mM iodoacetamide in 100 mM ammonium bicarbonate at room temperature for another 30 minutes. Subsequently, trypsin/lys C was added, incubated at 70°C for 1 hour, and quenched with 0.5% trifluoroacetic acid.

Following digestion, nano-liquid chromatography tandem mass spectrometry (Nano-LC/MS/MS) was performed using a Q Exactive HF Orbitrap mass spectrometer with an EASY Spray nanospray source (Thermo Scientific). The UltiMate™ 3000 RSLCnano LC system was used with water (0.1% formic acid) as Mobile Phase A and acetonitrile (0.1% formic acid) as Mobile Phase B. Chromatographic separations were carried out using a PharmaFluidics 50 cm mPAC™ column at 40°C. Peptides were eluted into the Q Exactive system using a gradient of Mobile Phase B from 1-20% over 100 minutes, 45% at minute 123, and 95% at minute 130.

MS/MS spectra were analyzed using Sequest™ (Proteome Discoverer 3.0.1.27, Thermo Fisher Scientific) against the Mus musculus proteome (NCBI TaxID:10090) with a fragment ion mass tolerance of 0.020 Da and a parent ion tolerance of 10.0 ppm. Fixed modification was set to cysteine carbamidomethylation, and variable modifications included methionine oxidation, methionine loss, and N-terminal acetylation. Protein abundance datasets were filtered to remove proteins with low or medium confidence determined by a false discovery rate threshold of 0.01. Filtered data containing high-confidence proteins from the colon were uploaded into the web-based MetaboAnalyst 6.0 tool. Missing values were substituted with 1/5 of the minimum values for each respective variable. To adjust differences among samples, the dataset was normalized by the median, transformed by log10, and then range scaled.

## Data Availability

The mass spectrometry proteomics data have been deposited to the ProteomeXchange Consortium via the PRIDE (52) partner repository with the dataset identifier PXD070273.

## Supplementary material

Supplementary Figure 1 Gene Set Enrichment Analysis (GSEA) plot evaluating enrichment of our curated set of genes related to mucus barrier disruption in Crohn’s Disease and ulcerative colitis. **(A)** GSEA assesses whether the predefined mucus barrier gene set of 96 genes is significantly enriched toward the top or bottom of a ranked list of genes differentially expressed in CD versus control or **(B)** UC vs control. In these plots, genes are ranked horizontally by their correlation with the disease phenotype by log fold change. The vertical black lines represent positions where genes from the mucus barrier gene set appear within this ranked list. The green enrichment profile line indicates a cumulative enrichment score showing how genes in the mucus barrier set are predominantly upregulated or downregulated in IBD. The significance of the observed enrichment is quantified by the normalized enrichment score (NES). This approach provides a way to test whether mucus-associated genes, considered collectively rather than one by one, show coordinated shifts in IBD tissue. By displaying the enrichment separately for CD and UC, the plots allow direct comparison of how strongly mucus barrier biology is perturbed in each subtype. These enrichment profiles support that mucus-related pathways exhibit a disease-linked signature across independent IBD patient datasets.

Supplementary Table 1 Total genes tested using Metavolcano to compare human colonic biopsies with Crohn’s disease against healthy controls.

Supplementary Table 2 Total genes tested using Metavolcano to compare human colonic biopsies with ulcerative colitis against healthy controls.

Supplementary Table 3 Total proteins tested comparing mouse colon tissue in *Agr2*^-/-^ mice with primary mucin secretory failure against healthy WT controls.
