# Supplementary figures and images for: Cross-species systems analysis distinguishes inflammatory remodeling from primary mucus secretory failure in inflammatory bowel disease
Source: Front Immunol. 2026 Jun 9;17:1813019. doi: 10.3389/fimmu.2026.1813019 (PMC13287023; doi:10.3389/fimmu.2026.1813019)

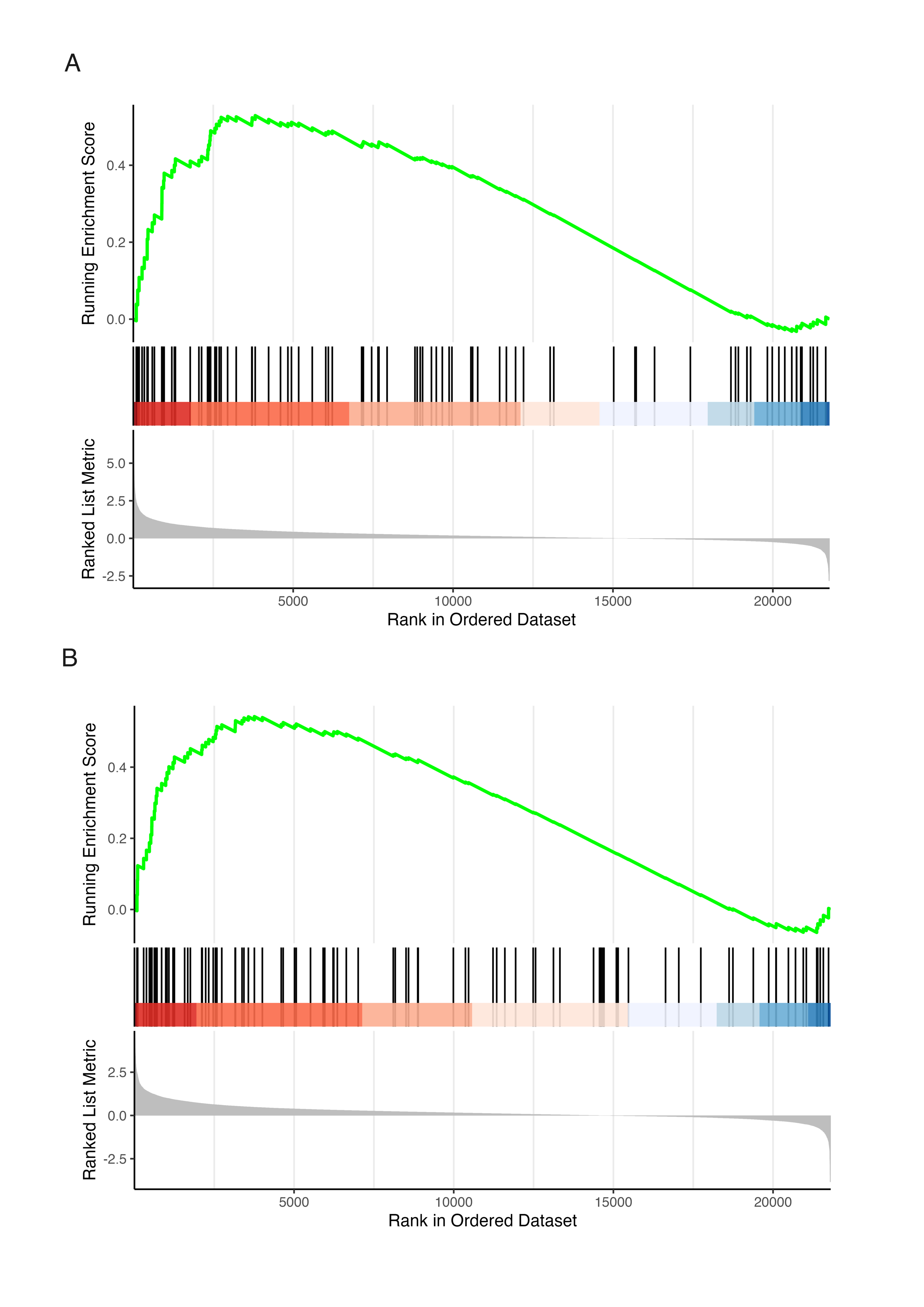

Supplement: Supplementary Figure 1 — Gene Set Enrichment Analysis (GSEA) plot evaluating enrichment of our curated set of genes related to mucus barrier disruption in Crohn’s Disease and ulcerative colitis. (A) GSEA assesses whether the predefined mucus barrier gene set of 96 genes is significantly enriched toward the top or bottom of a ranked list of genes differentially expressed in CD versus control or (B) UC vs control. In these plots, genes are ranked horizontally by their correlation with the disease phenotype by log fold change. The vertical black lines represent positions where genes from the mucus barrier gene set appear within this ranked list. The green enrichment profile line indicates a cumulative enrichment score showing how genes in the mucus barrier set are predominantly upregulated or downregulated in IBD. The significance of the observed enrichment is quantified by the normalized enrichment score (NES). This approach provides a way to test whether mucus-associated genes, considered collectively rather than one by one, show coordinated shifts in IBD tissue. By displaying the enrichment separately for CD and UC, the plots allow direct comparison of how strongly mucus barrier biology is perturbed in each subtype. These enrichment profiles support that mucus-related pathways exhibit a disease-linked signature across independent IBD patient datasets. [file Image1.tif]
