# Supplementary material for: Cross-species systems analysis distinguishes inflammatory remodeling from primary mucus secretory failure in inflammatory bowel disease
Source: Front Immunol. 2026 Jun 9;17:1813019. doi: 10.3389/fimmu.2026.1813019 (PMC13287023; doi:10.3389/fimmu.2026.1813019)
Supplement: Supplementary file 5 [file Table4.docx]

**SUPPLEMENTARY REFERENCES**

1. Park, Sung-Woo, Guohua Zhen, Catherine Verhaeghe, Yasuhiro Nakagami, Louis T. Nguyenvu, Andrea J. Barczak, Nigel Killeen, and David J. Erle. "The Protein Disulfide Isomerase AGR2 Is Essential for Production of Intestinal Mucus." Proceedings of the National Academy of Sciences 106, no. 17 (April 28, 2009): 6950–55. https://doi.org/10.1073/pnas.0808722106.
2. Al-Shaibi, Ahmad A., Ussama M. Abdel-Motal, Satanay Z. Hubrack, Alex N. Bullock, Amna A. Al-Marri, Nourhen Agrebi, Abdulrahman A. Al-Subaiey, et al. "Human AGR2 Deficiency Causes Mucus Barrier Dysfunction and Infantile Inflammatory Bowel Disease." *Cellular and Molecular Gastroenterology and Hepatology* 12, no. 5 (2021): 1809–30. https://doi.org/10.1016/j.jcmgh.2021.07.001.
3. Lamas, Bruno, Jane M. Natividad, and Harry Sokol. "Aryl Hydrocarbon Receptor and Intestinal Immunity." *Mucosal Immunology* 11, no. 4 (July 2018): 1024–38. <https://doi.org/10.1038/s41385-018-0019-2>.
4. Han, Huajun, Laurie A. Davidson, Yang-Yi Fan, Kerstin K. Landrock, Arul Jayaraman, Stephen H. Safe, and Robert S. Chapkin. "Loss of Aryl Hydrocarbon Receptor Suppresses the Response of Colonic Epithelial Cells to IL22 Signaling by Upregulating SOCS3." *American Journal of Physiology. Gastrointestinal and Liver Physiology* 322, no. 1 (January 1, 2022): G93–106. <https://doi.org/10.1152/ajpgi.00074.2021>.
5. Bjerknes, Matthew, and Hazel Cheng. "Cell Lineage Metastability in Gfi1-Deficient Mouse Intestinal Epithelium." *Developmental Biology* 345, no. 1 (September 2010): 49–63. <https://doi.org/10.1016/j.ydbio.2010.06.021>.
6. Lo, Yuan-Hung, Eunah Chung, Zhaohui Li, Ying-Wooi Wan, Maxime M. Mahe, Min-Shan Chen, Taeko K. Noah, et al. "Transcriptional Regulation by ATOH1 and Its Target SPDEF in the Intestine." *Cellular and Molecular Gastroenterology and Hepatology* 3, no. 1 (January 2017): 51–71. <https://doi.org/10.1016/j.jcmgh.2016.10.001>.
7. Lo, Yuan-Hung, Eunah Chung, Zhaohui Li, Ying-Wooi Wan, Maxime M. Mahe, Min-Shan Chen, Taeko K. Noah, et al. "Transcriptional Regulation by ATOH1 and Its Target SPDEF in the Intestine." *Cellular and Molecular Gastroenterology and Hepatology* 3, no. 1 (January 2017): 51–71. <https://doi.org/10.1016/j.jcmgh.2016.10.001>.
8. Thornberry, N. A., H. G. Bull, J. R. Calaycay, K. T. Chapman, A. D. Howard, M. J. Kostura, D. K. Miller, S. M. Molineaux, J. R. Weidner, and J. Aunins. "A Novel Heterodimeric Cysteine Protease Is Required for Interleukin-1 Beta Processing in Monocytes." *Nature* 356, no. 6372 (April 30, 1992): 768–74. <https://doi.org/10.1038/356768a0>.
9. Wlodarska, Marta, Christoph A. Thaiss, Roni Nowarski, Jorge Henao-Mejia, Jian-Ping Zhang, Eric M. Brown, Gad Frankel, et al. "NLRP6 Inflammasome Orchestrates the Colonic Host-Microbial Interface by Regulating Goblet Cell Mucus Secretion." *Cell*156, no. 5 (February 2014): 1045–59. <https://doi.org/10.1016/j.cell.2014.01.026>.
10. Brown, Rachel E., Justin Jacobse, Shruti A. Anant, Koral M. Blunt, Bob Chen, Paige N. Vega, Chase T. Jones, et al. "MTG16 (CBFA2T3) Regulates Colonic Epithelial Differentiation, Colitis, and Tumorigenesis by Repressing E Protein Transcription Factors." bioRxiv, February 16, 2022. <https://doi.org/10.1101/2021.11.03.467178>.
11. Mutoh, Hiroyuki, Hirotsugu Sakamoto, Hiroko Hayakawa, Yukitomo Arao, Kiichi Satoh, Mitsuhiro Nokubi, and Kentaro Sugano. "The Intestine-Specific Homeobox Gene Cdx2 Induces Expression of the Basic Helix–Loop–Helix Transcription Factor Math1." *Differentiation* 74, no. 6 (July 2006): 313–21. <https://doi.org/10.1111/j.1432-0436.2006.00074.x>.
12. Keller, Matthew S., Toshihiko Ezaki, Rong-Jun Guo, and John P. Lynch. "Cdx1 or Cdx2 Expression Activates E-Cadherin-Mediated Cell-Cell Adhesion and Compaction in Human COLO 205 Cells." *American Journal of Physiology-Gastrointestinal and Liver Physiology* 287, no. 1 (July 2004): G104–14. <https://doi.org/10.1152/ajpgi.00484.2003>.
13. Yang, Xiaofang, Ting Ye, Li Rong, Hong Peng, Jin Tong, Xiao Xiao, Xiaoqiang Wan, and Jinjun Guo. "GATA4 Forms a Positive Feedback Loop with CDX2 to Transactivate MUC2 in Bile Acids-Induced Gastric Intestinal Metaplasia." *Gut and Liver*, March 2, 2023. <https://doi.org/10.5009/gnl220394>.
14. Yu, Tianxin, Xi Chen, Wen Zhang, Juan Li, Ren Xu, Timothy C. Wang, Walden Ai, and Chunming Liu. "Krüppel-like Factor 4 Regulates Intestinal Epithelial Cell Morphology and Polarity." Edited by Neil A. Hotchin. *PLoS ONE* 7, no. 2 (February 24, 2012): e32492. <https://doi.org/10.1371/journal.pone.0032492>.
15. Shimada, Tadahito, Takero Koike, Michiko Yamagata, Masashi Yoneda, and Hideyuki Hiraishi. "Regulation of TFF3 Expression by Homeodomain Protein CDX2." *Regulatory Peptides* 140, no. 1 (April 5, 2007): 81–87. <https://doi.org/10.1016/j.regpep.2006.11.014>.
16. Blache, Philippe, Marc Van De Wetering, Isabelle Duluc, Claire Domon, Philippe Berta, Jean-Noël Freund, Hans Clevers, and Philippe Jay. "SOX9 Is an Intestine Crypt Transcription Factor, Is Regulated by the Wnt Pathway, and Represses the *CDX2* and *MUC2* Genes." *The Journal of Cell Biology* 166, no. 1 (July 5, 2004): 37–47. <https://doi.org/10.1083/jcb.200311021>.
17. Qin, Tingfeng, Jie Yang, Dayin Huang, Zhijun Zhang, Yanling Huang, Hui Chen, and Geyang Xu. "DOCK4 Stimulates MUC2 Production through Its Effect on Goblet Cell Differentiation." *Journal of Cellular Physiology* 236, no. 9 (September 2021): 6507–19. <https://doi.org/10.1002/jcp.30325>.
18. Qin, Tingfeng, Jie Yang, Dayin Huang, Zhijun Zhang, Yanling Huang, Hui Chen, and Geyang Xu. "DOCK4 Stimulates MUC2 Production through Its Effect on Goblet Cell Differentiation." *Journal of Cellular Physiology* 236, no. 9 (September 2021): 6507–19. <https://doi.org/10.1002/jcp.30325>.
19. Ng, Annie Y.-N., Paul Waring, Sika Ristevski, Caroline Wang, Trevor Wilson, Melanie Pritchard, Paul Hertzog, and Ismail Kola. "Inactivation of the Transcription Factor Elf3 in Mice Results in Dysmorphogenesis and Altered Differentiation of Intestinal Epithelium." *Gastroenterology* 122, no. 5 (May 2002): 1455–66. <https://doi.org/10.1053/gast.2002.32990>.
20. Kozutsumi, Yasunori, Mark Segal, Karl Normington, Mary-Jane Gething, and Joe Sambrook. "The Presence of Malfolded Proteins in the Endoplasmic Reticulum Signals the Induction of Glucose-Regulated Proteins." *Nature* 332, no. 6163 (March 1988): 462–64. <https://doi.org/10.1038/332462a0>.
21. Hasnain, Sumaira Z., Sharyn Tauro, Indrajit Das, Hui Tong, Alice C.–H. Chen, Penny L. Jeffery, Victoria McDonald, Timothy H. Florin, and Michael A. McGuckin. "IL-10 Promotes Production of Intestinal Mucus by Suppressing Protein Misfolding and Endoplasmic Reticulum Stress in Goblet Cells." *Gastroenterology* 144, no. 2 (February 2013): 357-368.e9. <https://doi.org/10.1053/j.gastro.2012.10.043>.
22. Calfon, Marcella, Huiqing Zeng, Fumihiko Urano, Jeffery H. Till, Stevan R. Hubbard, Heather P. Harding, Scott G. Clark, and David Ron. "IRE1 Couples Endoplasmic Reticulum Load to Secretory Capacity by Processing the XBP-1 mRNA." *Nature* 415, no. 6867 (January 3, 2002): 92–96. <https://doi.org/10.1038/415092a>.
23. Cloots, Eva, Mariska S. Simpson, Clint De Nolf, Wayne I. Lencer, Sophie Janssens, and Michael J. Grey. “Evolution and Function of the Epithelial Cell-Specific ER Stress Sensor IRE1β.” *Mucosal Immunology* 14, no. 6 (November 2021): 1235–46. https://doi.org/10.1038/s41385-021-00412-8.
24. Bell, Sheila M., Liqian Zhang, Yan Xu, Valerie Besnard, Susan E. Wert, Noah Shroyer, and Jeffrey A. Whitsett. "Kruppel-like Factor 5 Controls Villus Formation and Initiation of Cytodifferentiation in the Embryonic Intestinal Epithelium." *Developmental Biology* 375, no. 2 (March 2013): 128–39. <https://doi.org/10.1016/j.ydbio.2012.12.010>.
25. Lai, Yun-Ren, Yu-Fen Lu, Huang-Wei Lien, Chang-Jen Huang, and Sheng-Ping L. Hwang. "Foxa2 and Hif1ab Regulate Maturation of Intestinal Goblet Cells by Modulating *Agr2* Expression in Zebrafish Embryos." *Biochemical Journal* 473, no. 14 (July 15, 2016): 2205–18. <https://doi.org/10.1042/BCJ20160392>.
26. Ludikhuize, Marlies C., Maaike Meerlo, Marc Pages Gallego, Despina Xanthakis, Mar Burgaya Julià, Nguyen T.B. Nguyen, Eline C. Brombacher, et al. "Mitochondria Define Intestinal Stem Cell Differentiation Downstream of a FOXO/Notch Axis." *Cell Metabolism* 32, no. 5 (November 2020): 889-900.e7. <https://doi.org/10.1016/j.cmet.2020.10.005>.
27. Ludikhuize, Marlies C., Maaike Meerlo, Marc Pages Gallego, Despina Xanthakis, Mar Burgaya Julià, Nguyen T.B. Nguyen, Eline C. Brombacher, et al. "Mitochondria Define Intestinal Stem Cell Differentiation Downstream of a FOXO/Notch Axis." *Cell Metabolism* 32, no. 5 (November 2020): 889-900.e7. <https://doi.org/10.1016/j.cmet.2020.10.005>.
28. Kwon, Min-chul, Bon-Kyoung Koo, Yoon-Young Kim, Sang-Hee Lee, Nam-Shik Kim, Jae-Hwan Kim, and Young-Yun Kong. "Essential Role of CR6-Interacting Factor 1 (Crif1) in E74-like Factor 3 (ELF3)-Mediated Intestinal Development." *Journal of Biological Chemistry* 284, no. 48 (November 2009): 33634–41. <https://doi.org/10.1074/jbc.M109.059840>.
29. Gerbe, François, Johan H. Van Es, Leila Makrini, Bénédicte Brulin, Georg Mellitzer, Sylvie Robine, Béatrice Romagnolo, et al. “Distinct ATOH1 and Neurog3 Requirements Define Tuft Cells as a New Secretory Cell Type in the Intestinal Epithelium.” *Journal of Cell Biology* 192, no. 5 (March 7, 2011): 767–80. <https://doi.org/10.1083/jcb.201010127>.
30. Shroyer, Noah F., Deeann Wallis, Koen J.T. Venken, Hugo J. Bellen, and Huda Y. Zoghbi. "*Gfi1* Functions Downstream of *Math1* to Control Intestinal Secretory Cell Subtype Allocation and Differentiation." *Genes & Development* 19, no. 20 (October 15, 2005): 2412–17. <https://doi.org/10.1101/gad.1353905>.
31. Zhu, Liuluan, Qingcai Meng, Shuntao Liang, Yaluan Ma, Rui Li, Guoli Li, and Hui Zeng. "The Transcription Factor GFI1 Negatively Regulates NLRP3 Inflammasome Activation in Macrophages." *FEBS Letters* 588, no. 23 (November 28, 2014): 4513–19. <https://doi.org/10.1016/j.febslet.2014.10.025>.
32. Kim, Jae Hyeon, Jae Bum Ahn, Da Hye Kim, Soochan Kim, Hyun Woo Ma, Xiumei Che, Dong Hyuk Seo, et al. "Glutathione S‐transferase Theta 1 Protects against Colitis through Goblet Cell Differentiation via Interleukin‐22." *The FASEB Journal* 34, no. 2 (February 2020): 3289–3304. <https://doi.org/10.1096/fj.201902421R>
33. Kim, Jae Hyeon, Jae Bum Ahn, Da Hye Kim, Soochan Kim, Hyun Woo Ma, Xiumei Che, Dong Hyuk Seo, et al. "Glutathione S‐transferase Theta 1 Protects against Colitis through Goblet Cell Differentiation via Interleukin‐22." *The FASEB Journal* 34, no. 2 (February 2020): 3289–3304. <https://doi.org/10.1096/fj.201902421R>.
34. Zheng, Xiu, Kiichiro Tsuchiya, Ryuichi Okamoto, Michiko Iwasaki, Yoshihito Kano, Naoya Sakamoto, Tetsuya Nakamura, and Mamoru Watanabe. "Suppression of Hath1 Gene Expression Directly Regulated by Hes1 via Notch Signaling Is Associated with Goblet Cell Depletion in Ulcerative Colitis." *Inflammatory Bowel Diseases* 17, no. 11 (November 2011): 2251–60. <https://doi.org/10.1002/ibd.21611>.
35. Ghaleb, Amr M., Gaurav Aggarwal, Agnieszka B. Bialkowska, Mandayam O. Nandan, and Vincent W. Yang. "Notch Inhibits Expression of the Krüppel-Like Factor 4 Tumor Suppressor in the Intestinal Epithelium." *Molecular Cancer Research : MCR* 6, no. 12 (December 2008): 1920–27. <https://doi.org/10.1158/1541-7786.MCR-08-0224>.
36. Jensen, Jan, Erna Engholm Pedersen, Philip Galante, Jacob Hald, R. Scott Heller, Makoto Ishibashi, Ryoichiro Kageyama, Francois Guillemot, Palle Serup, and Ole D. Madsen. "Control of Endodermal Endocrine Development by Hes-1." *Nature Genetics* 24, no. 1 (January 2000): 36–44. <https://doi.org/10.1038/71657>.
37. Dowdell, Alexander S., Ian M. Cartwright, Matthew S. Goldberg, Rachael Kostelecky, Tyler Ross, Nichole Welch, Louis E. Glover, and Sean P. Colgan. "The HIF Target ATG9A Is Essential for Epithelial Barrier Function and Tight Junction Biogenesis." *Molecular Biology of the Cell* 31, no. 20 (September 15, 2020): 2249–58. <https://doi.org/10.1091/mbc.E20-05-0291>.
38. Dilly, Ashok K., Yong J. Lee, Herbert J. Zeh, Zong Sheng Guo, David L. Bartlett, and Haroon A. Choudry. "Targeting Hypoxia-Mediated Mucin 2 Production as a Therapeutic Strategy for Mucinous Tumors." *Translational Research* 169 (March 2016): 19-30.e1. <https://doi.org/10.1016/j.trsl.2015.10.006>.
39. Ramakrishnan, Sadeesh K., and Yatrik M. Shah. "Role of Intestinal HIF-2α in Health and Disease." *Annual Review of Physiology* 78, no. 1 (February 10, 2016): 301–25. <https://doi.org/10.1146/annurev-physiol-021115-105202>.
40. Hasnain, Sumaira Z., Sharyn Tauro, Indrajit Das, Hui Tong, Alice C.–H. Chen, Penny L. Jeffery, Victoria McDonald, Timothy H. Florin, and Michael A. McGuckin. "IL-10 Promotes Production of Intestinal Mucus by Suppressing Protein Misfolding and Endoplasmic Reticulum Stress in Goblet Cells." *Gastroenterology* 144, no. 2 (February 2013): 357-368.e9. <https://doi.org/10.1053/j.gastro.2012.10.043>.
41. Hasnain, Sumaira Z., Sharyn Tauro, Indrajit Das, Hui Tong, Alice C.–H. Chen, Penny L. Jeffery, Victoria McDonald, Timothy H. Florin, and Michael A. McGuckin. "IL-10 Promotes Production of Intestinal Mucus by Suppressing Protein Misfolding and Endoplasmic Reticulum Stress in Goblet Cells." *Gastroenterology* 144, no. 2 (February 2013): 357-368.e9. <https://doi.org/10.1053/j.gastro.2012.10.043>.
42. Cao, Hui, Jing Zhang, Hong Liu, Ledong Wan, Honghe Zhang, Qiong Huang, Enping Xu, and Maode Lai. "IL-13/STAT6 Signaling Plays a Critical Role in the Epithelial-Mesenchymal Transition of Colorectal Cancer Cells." *Oncotarget* 7, no. 38 (September 20, 2016): 61183–98. <https://doi.org/10.18632/oncotarget.11282>.
43. Lin, Xun, Stephen J. Gaudino, Kyung Ku Jang, Tej Bahadur, Ankita Singh, Anirban Banerjee, Michael Beaupre, et al. "IL-17RA-Signaling in Lgr5+ Intestinal Stem Cells Induces Expression of Transcription Factor ATOH1 to Promote Secretory Cell Lineage Commitment." *Immunity* 55, no. 2 (February 2022): 237-253.e8. <https://doi.org/10.1016/j.immuni.2021.12.016>.
44. Shi, Ning, Jing Zhang, and Shi-You Chen. “Runx2, a Novel Regulator for Goblet Cell Differentiation and Asthma Development.” *FASEB Journal: Official Publication of the Federation of American Societies for Experimental Biology* 31, no. 1 (January 2017): 412–20. https://doi.org/10.1096/fj.201600954R.
45. Kim, Jae Hyeon, Jae Bum Ahn, Da Hye Kim, Soochan Kim, Hyun Woo Ma, Xiumei Che, Dong Hyuk Seo, et al. "Glutathione S‐transferase Theta 1 Protects against Colitis through Goblet Cell Differentiation via Interleukin‐22." *The FASEB Journal* 34, no. 2 (February 2020): 3289–3304. <https://doi.org/10.1096/fj.201902421R>.
46. Sovran, Bruno, Linda M. P. Loonen, Peng Lu, Floor Hugenholtz, Clara Belzer, Ellen H. Stolte, Mark V. Boekschoten, et al. “IL-22-STAT3 Pathway Plays a Key Role in the Maintenance of Ileal Homeostasis in Mice Lacking Secreted Mucus Barrier:” *Inflammatory Bowel Diseases* 21, no. 3 (March 2015): 531–42. <https://doi.org/10.1097/MIB.0000000000000319>.
47. Mahapatro, Mousumi, Sebastian Foersch, Manuela Hefele, Gui-Wei He, Elisa Giner-Ventura, Tamar Mchedlidze, Markus Kindermann, et al. “Programming of Intestinal Epithelial Differentiation by IL-33 Derived from Pericryptal Fibroblasts in Response to Systemic Infection.” *Cell Reports* 15, no. 8 (May 2016): 1743–56. <https://doi.org/10.1016/j.celrep.2016.04.049>.
48. Li, Yanrong, Yujie Jia, Tingfang Cui, and Jiayuan Zhang. "IL‑6/STAT3 Signaling Pathway Regulates the Proliferation and Damage of Intestinal Epithelial Cells in Patients with Ulcerative Colitis via H3K27ac." *Experimental and Therapeutic Medicine*22, no. 2 (June 17, 2021): 890. <https://doi.org/10.3892/etm.2021.10322>.
49. Cantero-Recasens, Gerard, Cristian M Butnaru, Miguel A Valverde, José R Naranjo, Nathalie Brouwers, and Vivek Malhotra. "KChIP3 Coupled to Ca2+ Oscillations Exerts a Tonic Brake on Baseline Mucin Release in the Colon." *eLife* 7 (October 1, 2018): e39729. <https://doi.org/10.7554/eLife.39729>.
50. Baird, Liam, and Masayuki Yamamoto. "The Molecular Mechanisms Regulating the KEAP1-NRF2 Pathway." *Molecular and Cellular Biology* 40, no. 13 (June 15, 2020): e00099-20. <https://doi.org/10.1128/MCB.00099-20>.
51. Katz, Jonathan P., Nathalie Perreault, Bree G. Goldstein, Catherine S. Lee, Patricia A. Labosky, Vincent W. Yang, and Klaus H. Kaestner. "The Zinc-Finger Transcription Factor Klf4 Is Required for Terminal Differentiation of Goblet Cells in the Colon." *Development (Cambridge, England)* 129, no. 11 (June 2002): 2619–28. <https://www.ncbi.nlm.nih.gov/pmc/articles/PMC2225535/>.
52. Yori, Jennifer L., Emhonta Johnson, Guangjin Zhou, Mukesh K. Jain, and Ruth A. Keri. "Krüppel-like Factor 4 Inhibits Epithelial-to-Mesenchymal Transition through Regulation of E-Cadherin Gene Expression." *The Journal of Biological Chemistry*285, no. 22 (May 28, 2010): 16854–63. <https://doi.org/10.1074/jbc.M110.114546>.
53. Bell, Sheila M., Liqian Zhang, Yan Xu, Valerie Besnard, Susan E. Wert, Noah Shroyer, and Jeffrey A. Whitsett. "Kruppel-like Factor 5 Controls Villus Formation and Initiation of Cytodifferentiation in the Embryonic Intestinal Epithelium." *Developmental Biology* 375, no. 2 (March 2013): 128–39. <https://doi.org/10.1016/j.ydbio.2012.12.010>.
54. Grinat, Johanna, Frauke Kosel, Neha Goveas, Andrea Kranz, Dimitra Alexopoulou, Klaus Rajewsky, Michael Sigal, A Francis Stewart, and Julian Heuberger. "Epigenetic Modifier Balances Mapk and Wnt Signalling in Differentiation of Goblet and Paneth Cells." *Life Science Alliance* 5, no. 4 (April 2022): e202101187. <https://doi.org/10.26508/lsa.202101187>.
55. Gonzalez-Perez, Vivian, Pedro L. Martinez-Espinosa, Monica Sala-Rabanal, Nikhil Bharadwaj, Xiao-Ming Xia, Albert C. Chen, David Alvarado, et al. "Goblet Cell LRRC26 Regulates BK Channel Activation and Protects against Colitis in Mice." *Proceedings of the National Academy of Sciences* 118, no. 3 (January 19, 2021): e2019149118. <https://doi.org/10.1073/pnas.2019149118>.
56. Li, Yuehua, Linda D. Martin, Gwendolyn Spizz, and Kenneth B. Adler. "MARCKS Protein Is a Key Molecule Regulating Mucin Secretion by Human Airway Epithelial Cells in Vitro." *Journal of Biological Chemistry* 276, no. 44 (November 2001): 40982–90. <https://doi.org/10.1074/jbc.M105614200>.
57. Schütte, André, Anna Ermund, Christoph Becker-Pauly, Malin E. V. Johansson, Ana M. Rodriguez-Pineiro, Fredrik Bäckhed, Stefan Müller, Daniel Lottaz, Judith S. Bond, and Gunnar C. Hansson. “Microbial-Induced Meprin β Cleavage in MUC2 Mucin and a Functional CFTR Channel Are Required to Release Anchored Small Intestinal Mucus.” *Proceedings of the National Academy of Sciences* 111, no. 34 (August 26, 2014): 12396–401. <https://doi.org/10.1073/pnas.1407597111>.
58. Pereira, Bruno, Sofia Sousa, Rita Barros, Laura Carreto, Patrícia Oliveira, Carla Oliveira, Nicolas T. Chartier, et al. “CDX2 Regulation by the RNA-Binding Protein MEX3A: Impact on Intestinal Differentiation and Stemness.” *Nucleic Acids Research*41, no. 7 (April 2013): 3986–99. <https://doi.org/10.1093/nar/gkt087>.
59. Garg, Pallavi, Anupama Ravi, Neal R. Patel, Jesse Roman, Andrew T. Gewirtz, Didier Merlin, and Shanthi V. Sitaraman. "Matrix Metalloproteinase-9 Regulates MUC-2 Expression Through Its Effect on Goblet Cell Differentiation." *Gastroenterology* 132, no. 5 (May 1, 2007): 1877–89. <https://doi.org/10.1053/j.gastro.2007.02.048>.
60. Zhou, Y., P. Rychahou, Q. Wang, H. L. Weiss, and B. M. Evers. "TSC2/mTORC1 Signaling Controls Paneth and Goblet Cell Differentiation in the Intestinal Epithelium." *Cell Death & Disease* 6, no. 2 (February 2015): e1631–e1631. <https://doi.org/10.1038/cddis.2014.588>.
61. Frantz, A L, E W Rogier, C R Weber, L Shen, D A Cohen, L A Fenton, M E C Bruno, and C S Kaetzel. "Targeted Deletion of MyD88 in Intestinal Epithelial Cells Results in Compromised Antibacterial Immunity Associated with Downregulation of Polymeric Immunoglobulin Receptor, Mucin-2, and Antibacterial Peptides." *Mucosal Immunology* 5, no. 5 (September 2012): 501–12. <https://doi.org/10.1038/mi.2012.23>.
62. Jenny, M. "Neurogenin3 Is Differentially Required for Endocrine Cell Fate Specification in the Intestinal and Gastric Epithelium." *The EMBO Journal* 21, no. 23 (December 1, 2002): 6338–47. <https://doi.org/10.1093/emboj/cdf649>.
63. Wang, Q., Y. Zhou, P. Rychahou, C. Liu, H. L. Weiss, and B. M. Evers. "NFAT5 Represses Canonical Wnt Signaling via Inhibition of β -Catenin Acetylation and Participates in Regulating Intestinal Cell Differentiation." *Cell Death & Disease* 4, no. 6 (June 2013): e671–e671. <https://doi.org/10.1038/cddis.2013.202>.
64. Yagishita, Yoko, Melissa L. McCallum, Thomas W. Kensler, and Nobunao Wakabayashi. "Constitutive Activation of Nrf2 in Mice Expands Enterogenesis in Small Intestine Through Negative Regulation of Math1." *Cellular and Molecular Gastroenterology and Hepatology* 11, no. 2 (2021): 503–24. <https://doi.org/10.1016/j.jcmgh.2020.08.013>.
65. Loncar, M. B., E.-d Al-azzeh, P. S. M. Sommer, M. Marinovic, K. Schmehl, M. Kruschewski, N. Blin, R. Stohwasser, P. Gött, and T. Kayademir. "Tumour Necrosis Factor α and Nuclear Factor κB Inhibit Transcription of Human TFF3 Encoding a Gastrointestinal Healing Peptide." *Gut* 52, no. 9 (September 1, 2003): 1297–1303. <https://doi.org/10.1136/gut.52.9.1297>.
66. Iwashita, Jun, Yukita Sato, Hiroko Sugaya, Nagatomo Takahashi, Hiroshi Sasaki, and Tatsuya Abe. "mRNA of MUC2 Is Stimulated by IL‐4, IL‐13 or TNF‐α through a Mitogen‐activated Protein Kinase Pathway in Human Colon Cancer Cells." *Immunology & Cell Biology* 81, no. 4 (August 2003): 275–82. <https://doi.org/10.1046/j.1440-1711.2003.t01-1-01163.x>.
67. Xia, Bing, Ruqing Zhong, Weida Wu, Chengzeng Luo, Qingshi Meng, Qingtao Gao, Yong Zhao, et al. "Mucin O-Glycan-Microbiota Axis Orchestrates Gut Homeostasis in a Diarrheal Pig Model." *Microbiome* 10, no. 1 (August 31, 2022): 139. <https://doi.org/10.1186/s40168-022-01326-8>.
68. Venuprasad, K., and Arianne L. Theiss. "NLRP6 in Host Defense and Intestinal Inflammation." *Cell Reports* 35, no. 4 (April 2021): 109043. <https://doi.org/10.1016/j.celrep.2021.109043>.
69. Ferrand, Audrey, Ziad Al Nabhani, Núria Solà Tapias, Emmanuel Mas, Jean-Pierre Hugot, and Frédérick Barreau. "NOD2 Expression in Intestinal Epithelial Cells Protects Toward the Development of Inflammation and Associated Carcinogenesis." *Cellular and Molecular Gastroenterology and Hepatology* 7, no. 2 (2019): 357–69. <https://doi.org/10.1016/j.jcmgh.2018.10.009>.
70. Iso, Tatsuya, Larry Kedes, and Yasuo Hamamori. "HES and HERP Families: Multiple Effectors of the Notch Signaling Pathway." *Journal of Cellular Physiology* 194, no. 3 (March 2003): 237–55. <https://doi.org/10.1002/jcp.10208>.
71. Coant, Nicolas, Sanae Ben Mkaddem, Eric Pedruzzi, Cécile Guichard, Xavier Tréton, Robert Ducroc, Jean-Noel Freund, et al. "NADPH Oxidase 1 Modulates WNT and NOTCH1 Signaling To Control the Fate of Proliferative Progenitor Cells in the Colon." *Molecular and Cellular Biology* 30, no. 11 (June 1, 2010): 2636–50. <https://doi.org/10.1128/MCB.01194-09>.
72. Yagishita, Yoko, Melissa L. McCallum, Thomas W. Kensler, and Nobunao Wakabayashi. "Constitutive Activation of Nrf2 in Mice Expands Enterogenesis in Small Intestine Through Negative Regulation of Math1." *Cellular and Molecular Gastroenterology and Hepatology* 11, no. 2 (2021): 503–24. <https://doi.org/10.1016/j.jcmgh.2020.08.013>.
73. Shi, Ning, Jing Zhang, and Shi-You Chen. “Runx2, a Novel Regulator for Goblet Cell Differentiation and Asthma Development.” *FASEB Journal: Official Publication of the Federation of American Societies for Experimental Biology* 31, no. 1 (January 2017): 412–20. https://doi.org/10.1096/fj.201600954R.
74. Gagné-Sansfacon, Jessica, Ariane Langlois, Marie-Josée Langlois, Geneviève Coulombe, Sarah Tremblay, Vanessa Vaillancourt-Lavigueur, Cheng-Kui Qu, Alfredo Menendez, and Nathalie Rivard. “The Tyrosine Phosphatase Shp-2 Confers Resistance to Colonic Inflammation by Driving Goblet Cell Function and Crypt Regeneration: Shp-2 Protects the Intestinal Epithelium against Inflammation.” *The Journal of Pathology* 247, no. 1 (January 2019): 135–46. <https://doi.org/10.1002/path.5177>.
75. Daulagala, Amanda C., Mary Catherine Bridges, and Antonis Kourtidis. "E-Cadherin Beyond Structure: A Signaling Hub in Colon Homeostasis and Disease." *International Journal of Molecular Sciences* 20, no. 11 (June 5, 2019): 2756. <https://doi.org/10.3390/ijms20112756>.
76. Baulies, Anna, Nikolaos Angelis, Valentina Foglizzo, E. Thomas Danielsen, Harshil Patel, Laura Novellasdemunt, Anna Kucharska, et al. “The Transcription Co-Repressors MTG8 and MTG16 Regulate Exit of Intestinal Stem Cells From Their Niche and Differentiation Into Enterocyte vs Secretory Lineages.” *Gastroenterology*159, no. 4 (October 2020): 1328-1341.e3. <https://doi.org/10.1053/j.gastro.2020.06.012>.
77. Li, Chang, Yuning Zhou, Piotr Rychahou, Heidi L. Weiss, Eun Y. Lee, Courtney L. Perry, Terrence A. Barrett, Qingding Wang, and B. Mark Evers. "SIRT2 Contributes to the Regulation of Intestinal Cell Proliferation and Differentiation." *Cellular and Molecular Gastroenterology and Hepatology* 10, no. 1 (2020): 43–57. <https://doi.org/10.1016/j.jcmgh.2020.01.004>.
78. Han, Huajun, Laurie A. Davidson, Yang-Yi Fan, Kerstin K. Landrock, Arul Jayaraman, Stephen H. Safe, and Robert S. Chapkin. "Loss of Aryl Hydrocarbon Receptor Suppresses the Response of Colonic Epithelial Cells to IL22 Signaling by Upregulating SOCS3." *American Journal of Physiology-Gastrointestinal and Liver Physiology* 322, no. 1 (January 1, 2022): G93–106. <https://doi.org/10.1152/ajpgi.00074.2021>.
79. Blache, Philippe, Marc Van De Wetering, Isabelle Duluc, Claire Domon, Philippe Berta, Jean-Noël Freund, Hans Clevers, and Philippe Jay. "SOX9 Is an Intestine Crypt Transcription Factor, Is Regulated by the Wnt Pathway, and Represses the *CDX2* and *MUC2* Genes." *The Journal of Cell Biology* 166, no. 1 (July 5, 2004): 37–47. <https://doi.org/10.1083/jcb.200311021>.
80. Blache, Philippe, Marc Van De Wetering, Isabelle Duluc, Claire Domon, Philippe Berta, Jean-Noël Freund, Hans Clevers, and Philippe Jay. "SOX9 Is an Intestine Crypt Transcription Factor, Is Regulated by the Wnt Pathway, and Represses the *CDX2* and *MUC2* Genes." *The Journal of Cell Biology* 166, no. 1 (July 5, 2004): 37–47. <https://doi.org/10.1083/jcb.200311021>.
81. Gregorieff, Alex, Daniel E. Stange, Pekka Kujala, Harry Begthel, Maaike Van Den Born, Jeroen Korving, Peter J. Peters, and Hans Clevers. “The Ets-Domain Transcription Factor Spdef Promotes Maturation of Goblet and Paneth Cells in the Intestinal Epithelium.” *Gastroenterology* 137, no. 4 (October 2009): 1333-1345.e3. <https://doi.org/10.1053/j.gastro.2009.06.044>.
82. Noah, Taeko K., Avedis Kazanjian, Jeffrey Whitsett, and Noah F. Shroyer. "SAM Pointed Domain ETS Factor (SPDEF) Regulates Terminal Differentiation and Maturation of Intestinal Goblet Cells." *Experimental Cell Research* 316, no. 3 (February 2010): 452–65. <https://doi.org/10.1016/j.yexcr.2009.09.020>.
83. Sovran, Bruno, Linda M. P. Loonen, Peng Lu, Floor Hugenholtz, Clara Belzer, Ellen H. Stolte, Mark V. Boekschoten, et al. “IL-22-STAT3 Pathway Plays a Key Role in the Maintenance of Ileal Homeostasis in Mice Lacking Secreted Mucus Barrier:” *Inflammatory Bowel Diseases* 21, no. 3 (March 2015): 531–42. <https://doi.org/10.1097/MIB.0000000000000319>.
84. Schubart, Christoph, Branislav Krljanac, Manuel Otte, Cornelia Symowski, Eva Martini, Claudia Günther, Christoph Becker, Christoph Daniel, and David Voehringer. "Selective Expression of Constitutively Activated STAT6 in Intestinal Epithelial Cells Promotes Differentiation of Secretory Cells and Protection against Helminths." *Mucosal Immunology* 12, no. 2 (March 2019): 413–24. <https://doi.org/10.1038/s41385-018-0107-3>.
85. Gao, Yajing, Yan Yan, Sushil Tripathi, Nalle Pentinmikko, Ana Amaral, Pekka Päivinen, Eva Domènech-Moreno, et al. “LKB1 Represses ATOH1 via PDK4 and Energy Metabolism and Regulates Intestinal Stem Cell Fate.” *Gastroenterology* 158, no. 5 (April 2020): 1389-1401.e10. <https://doi.org/10.1053/j.gastro.2019.12.033>.
86. Aihara, Eitaro, Kristen A. Engevik, and Marshall H. Montrose. "Trefoil Factor Peptides and Gastrointestinal Function." *Annual Review of Physiology* 79, no. 1 (February 10, 2017): 357–80. <https://doi.org/10.1146/annurev-physiol-021115-105447>.
87. Iwashita, Jun, Yukita Sato, Hiroko Sugaya, Nagatomo Takahashi, Hiroshi Sasaki, and Tatsuya Abe. "mRNA of MUC2 Is Stimulated by IL‐4, IL‐13 or TNF‐α through a Mitogen‐activated Protein Kinase Pathway in Human Colon Cancer Cells." *Immunology & Cell Biology* 81, no. 4 (August 2003): 275–82. <https://doi.org/10.1046/j.1440-1711.2003.t01-1-01163.x>.
88. Loncar, M. B., E.-d Al-azzeh, P. S. M. Sommer, M. Marinovic, K. Schmehl, M. Kruschewski, N. Blin, R. Stohwasser, P. Gött, and T. Kayademir. "Tumour Necrosis Factor α and Nuclear Factor κB Inhibit Transcription of Human TFF3 Encoding a Gastrointestinal Healing Peptide." *Gut* 52, no. 9 (September 1, 2003): 1297–1303. <https://doi.org/10.1136/gut.52.9.1297>.
89. Cornick, Steve, Manish Kumar, France Moreau, Herbert Gaisano, and Kris Chadee. "VAMP8-Mediated MUC2 Mucin Exocytosis from Colonic Goblet Cells Maintains Innate Intestinal Homeostasis." *Nature Communications* 10, no. 1 (September 20, 2019): 4306. <https://doi.org/10.1038/s41467-019-11811-8>.
90. Guardavaccaro, Daniele, and Hans Clevers. "Wnt/β-Catenin and MAPK Signaling: Allies and Enemies in Different Battlefields." *Science Signaling* 5, no. 219 (April 10, 2012). <https://doi.org/10.1126/scisignal.2002921>.
91. Stengel, Stephanie T., Antonella Fazio, Simone Lipinski, Martin T. Jahn, Konrad Aden, Go Ito, Felix Wottawa, et al. "Activating Transcription Factor 6 Mediates Inflammatory Signals in Intestinal Epithelial Cells Upon Endoplasmic Reticulum Stress." *Gastroenterology* 159, no. 4 (October 2020): 1357-1374.e10. <https://doi.org/10.1053/j.gastro.2020.06.088>.
92. Kaser, Arthur, and Richard S. Blumberg. "Endoplasmic Reticulum Stress in the Intestinal Epithelium and Inflammatory Bowel Disease." *Seminars in Immunology* 21, no. 3 (June 2009): 156–63. <https://doi.org/10.1016/j.smim.2009.01.001>.
93. Bergström, Joakim H., George M. H. Birchenough, Gergely Katona, Bjoern O. Schroeder, André Schütte, Anna Ermund, Malin E. V. Johansson, and Gunnar C. Hansson. “Gram-Positive Bacteria Are Held at a Distance in the Colon Mucus by the Lectin-like Protein ZG16.” *Proceedings of the National Academy of Sciences* 113, no. 48 (November 29, 2016): 13833–38. <https://doi.org/10.1073/pnas.1611400113>.
94. Grey, Michael J., Heidi De Luca, Doyle V. Ward, Irini A.M. Kreulen, Katlynn Bugda Gwilt, Sage E. Foley, Jay R. Thiagarajah, Beth A. McCormick, Jerrold R. Turner, and Wayne I. Lencer. “The Epithelial-Specific ER Stress Sensor ERN2/IRE1β Enables Host-Microbiota Crosstalk to Affect Colon Goblet Cell Development.” *The Journal of Clinical Investigation* 132, no. 17: e153519. Accessed March 27, 2025. https://doi.org/10.1172/JCI153519.
95. Wandall, Hans H., Helle Hassan, Ekaterina Mirgorodskaya, Anne K. Kristensen, Peter Roepstorff, Eric P. Bennett, Peter A. Nielsen, et al. "Substrate Specificities of Three Members of the Human UDP-N-Acetyl-α-d-Galactosamine:Polypeptide N-Acetylgalactosaminyltransferase Family, GalNAc-T1, -T2, and -T3." *Journal of Biological Chemistry* 272, no. 38 (September 1997): 23503–14. <https://doi.org/10.1074/jbc.272.38.23503>.
96. Wandall, Hans H., Helle Hassan, Ekaterina Mirgorodskaya, Anne K. Kristensen, Peter Roepstorff, Eric P. Bennett, Peter A. Nielsen, et al. "Substrate Specificities of Three Members of the Human UDP-N-Acetyl-α-d-Galactosamine:Polypeptide N-Acetylgalactosaminyltransferase Family, GalNAc-T1, -T2, and -T3." *Journal of Biological Chemistry* 272, no. 38 (September 1997): 23503–14. <https://doi.org/10.1074/jbc.272.38.23503>.
97. Wandall, Hans H., Helle Hassan, Ekaterina Mirgorodskaya, Anne K. Kristensen, Peter Roepstorff, Eric P. Bennett, Peter A. Nielsen, et al. "Substrate Specificities of Three Members of the Human UDP-N-Acetyl-α-d-Galactosamine:Polypeptide N-Acetylgalactosaminyltransferase Family, GalNAc-T1, -T2, and -T3." *Journal of Biological Chemistry* 272, no. 38 (September 1997): 23503–14. <https://doi.org/10.1074/jbc.272.38.23503>.
98. Bennett, Eric Paul, Helle Hassan, Ulla Mandel, Ekatarina Mirgorodskaya, Peter Roepstorff, Joy Burchell, Joyce Taylor-Papadimitriou, et al. "Cloning of a Human UDP-N-Acetyl-α-d-Galactosamine:PolypeptideN-Acetylgalactosaminyltransferase That Complements Other GalNAc-Transferases in Complete O-Glycosylation of the MUC1 Tandem Repeat." *Journal of Biological Chemistry* 273, no. 46 (November 1998): 30472–81. <https://doi.org/10.1074/jbc.273.46.30472>.
99. Wu, Yi, Yafei Li, Zheng Ruan, Jiaojiao Li, Li Zhang, Hui Lu, and Zhenjiang Xu. "Puerarin Rebuilding the Mucus Layer and Regulating Mucin-Utilizing Bacteria to Relieve Ulcerative Colitis." *Journal of Agricultural and Food Chemistry* 68, no. 41 (October 14, 2020): 11402–11. <https://doi.org/10.1021/acs.jafc.0c04119>.
100. Bennett, Eric Paul, Helle Hassan, Michael A. Hollingsworth, and Henrik Clausen. "A Novel Human UDP- *N* -Acetyl- D -Galactosamine:Polypeptide *N* -Acetylgalactosaminyltransferase, GalNAc-T7, with Specificity for Partial GalNAc-Glycosylated Acceptor Substrates." *FEBS Letters* 460, no. 2 (October 29, 1999): 226–30. <https://doi.org/10.1016/S0014-5793(99)01268-5>.
101. Ong, Gideon, Rosemund Ragetli, Katarzyna Mnich, Bradley W. Doble, Wafa Kammouni, and Susan E. Logue. “IRE1 Signaling Increases PERK Expression during Chronic ER Stress.” *Cell Death & Disease* 15, no. 4 (April 18, 2024): 1–11. https://doi.org/10.1038/s41419-024-06663-0.
102. Evans, Daniel R., Srividya Venkitachalam, Leslie Revoredo, Amanda T. Dohey, Erica Clarke, Julia J. Pennell, Amy E. Powell, et al. “Evidence for GALNT12 as a Moderate Penetrance Gene for Colorectal Cancer.” *Human Mutation* 39, no. 8 (August 2018): 1092–1101. <https://doi.org/10.1002/humu.23549>.
103. Tang, Xuelian, Weijun Wang, Gaichao Hong, Caihan Duan, Siran Zhu, Yuen Tian, Chaoqun Han, Wei Qian, Rong Lin, and Xiaohua Hou. "Gut Microbiota-Mediated Lysophosphatidylcholine Generation Promotes Colitis in Intestinal Epithelium-Specific Fut2 Deficiency." *Journal of Biomedical Science* 28, no. 1 (March 15, 2021): 20. <https://doi.org/10.1186/s12929-021-00711-z>.
104. Cantero-Recasens, Gerard, Carla Burballa, Yuki Ohkawa, Tomohiko Fukuda, Yoichiro Harada, IBD Character Consortium, Amy J. Curwin, et al. "The Ulcerative Colitis-Associated Gene FUT8 Regulates the Quantity and Quality of Secreted Mucins." *Proceedings of the National Academy of Sciences* 119, no. 43 (October 25, 2022): e2205277119. <https://doi.org/10.1073/pnas.2205277119>.
105. Xiang, Ting, Muchuan Qiao, Jiangbo Xie, Zheng Li, and Hailong Xie. "Emerging Roles of the Unique Molecular Chaperone Cosmc in the Regulation of Health and Disease." *Biomolecules* 12, no. 12 (November 23, 2022): 1732. <https://doi.org/10.3390/biom12121732>.
106. Iwai, Toshie, Niro Inaba, Andreas Naundorf, Yan Zhang, Masanori Gotoh, Hiroko Iwasaki, Takashi Kudo, et al. "Molecular Cloning and Characterization of a Novel UDP-GlcNAc:GalNAc-Peptide Β1,3-N-Acetylglucosaminyltransferase (β3Gn-T6), an Enzyme Synthesizing the Core 3 Structure of O-Glycans∗." *Journal of Biological Chemistry* 277, no. 15 (April 12, 2002): 12802–9. <https://doi.org/10.1074/jbc.M112457200>.
107. Iwai, Toshie, Niro Inaba, Andreas Naundorf, Yan Zhang, Masanori Gotoh, Hiroko Iwasaki, Takashi Kudo, et al. "Molecular Cloning and Characterization of a Novel UDP-GlcNAc:GalNAc-Peptide Β1,3-N-Acetylglucosaminyltransferase (β3Gn-T6), an Enzyme Synthesizing the Core 3 Structure of O-Glycans∗." *Journal of Biological Chemistry* 277, no. 15 (April 12, 2002): 12802–9. <https://doi.org/10.1074/jbc.M112457200>.
108. Khiaowichit, Juthamas, Chutima Talabnin, Chavaboon Dechsukhum, Atit Silsirivanit, and Krajang Talabnin. "Down-Regulation of C1GALT1 Enhances the Progression of Cholangiocarcinoma through Activation of AKT/ERK Signaling Pathways." *Life* 12, no. 2 (January 25, 2022): 174. <https://doi.org/10.3390/life12020174>.
109. Pietrobono, Silvia, Giulia Anichini, Cesare Sala, Fabrizio Manetti, Luciana L. Almada, Sara Pepe, Ryan M. Carr, et al. "ST3GAL1 Is a Target of the SOX2-GLI1 Transcriptional Complex and Promotes Melanoma Metastasis through AXL." *Nature Communications* 11, no. 1 (November 17, 2020): 5865. <https://doi.org/10.1038/s41467-020-19575-2>.
110. Sánchez‐Martínez, Elisa, Manuel Garrido‐Romero, and Francisco Javier Moreno. "Functional Role of ST6GALNAC1 ‐mediated Sialylation of Mucins in Preserving Intestinal Barrier Integrity and Ameliorating Inflammation." *Allergy* 77, no. 12 (December 2022): 3697–98. <https://doi.org/10.1111/all.15489>.
111. Shaoul, Ron, Yoshio Okada, Ernest Cutz, and Margaret A. Marcon. "Colonic Expression of MUC2, MUC5AC, and TFF1 in Inflammatory Bowel Disease in Children." *Journal of Pediatric Gastroenterology and Nutrition* 38, no. 5 (May 2004): 488. <https://journals.lww.com/jpgn/Fulltext/2004/05000/Colonic_Expression_of_MUC2,_MUC5AC,_and_TFF1_in.6.aspx>.
112. Carroll, Daniela J., Mary W.N. Burns, Lynda Mottram, Daniel C. Propheter, Andrew Boucher, Gabrielle M. Lessen, Ashwani Kumar, et al. "Interleukin-22 Regulates B3GNT7 Expression to Induce Fucosylation of Glycoproteins in Intestinal Epithelial Cells." *Journal of Biological Chemistry* 298, no. 2 (February 2022): 101463. <https://doi.org/10.1016/j.jbc.2021.101463>.
113. Kanoh, Akira, Hideyuki Takeuchi, Kentaro Kato, Michihiko Waki, Katsuaki Usami, and Tatsuro Irimura. "Interleukin-4 Induces Specific Pp-GalNAc-T Expression and Alterations in Mucin O-Glycosylation in Colonic Epithelial Cells." *Biochimica et Biophysica Acta (BBA) - General Subjects* 1780, no. 3 (March 2008): 577–84. <https://doi.org/10.1016/j.bbagen.2007.08.004>.
